# High Resolution UHPLC-MS Metabolomics and Sedative-Anxiolytic Effects of *Latua pubiflora*: A Mystic Plant used by Mapuche Amerindians

**DOI:** 10.3389/fphar.2017.00494

**Published:** 2017-07-26

**Authors:** Eliana L. Sánchez-Montoya, Marco A. Reyes, Joel Pardo, Juana Nuñez-Alarcón, José G. Ortiz, Juan C. Jorge, Jorge Bórquez, Andrei Mocan, Mario J. Simirgiotis

**Affiliations:** ^1^Facultad de Ciencias, Instituto de Farmacia, Universidad Austral de Chile Valdivia, Chile; ^2^Facultad de Ciencias, Instituto de Química, Universidad Austral de Chile Valdivia, Chile; ^3^Department of Pharmacology, University of Puerto Rico San Juan, Puerto Rico; ^4^Department of Anatomy, University of Puerto Rico San Juan, Puerto Rico; ^5^Laboratorio de Productos Naturales, Departamento de Química, Facultad de Ciencias Básicas, Universidad de Antofagasta Antofagasta, Chile; ^6^Department of Pharmaceutical Botany, Faculty of Pharmacy, University of Medicine and Pharmacy “Iuliu Haţieganu” Cluj-Napoca, Romania; ^7^ICHAT and Institute for Life Sciences, University of Agricultural Sciences and Veterinary Medicine Cluj-Napoca, Romania; ^8^Center for Interdisciplinary Studies on the Nervous System, Universidad Austral de Chile Valdivia, Chile

**Keywords:** *Palo brujo*, psychoactive plant, scopolamine, UHPLC, metabolomics, *Latua publiflora* (Griseb) Phil.

## Abstract

*Latua pubiflora* (Griseb) Phil. Is a native shrub of the Solanaceae family that grows freely in southern Chile and is employed among Mapuche aboriginals to induce sedative effects and hallucinations in religious or medicine rituals since prehispanic times. In this work, the pentobarbital-induced sleeping test and the elevated plus maze test were employed to test the behavioral effects of extracts of this plant in mice. The psychopharmacological evaluation of *L. pubiflora* extracts in mice determined that both alkaloid-enriched as well as the non-alkaloid extracts produced an increase of sleeping time and alteration of motor activity in mice at 150 mg/Kg. The alkaloid extract exhibited anxiolytic effects in the elevated plus maze test, which was counteracted by flumazenil. In addition, the alkaloid extract from *L. pubiflora* decreased [^3^H]-flunitrazepam binding on rat cortical membranes. In this study we have identified 18 tropane alkaloids (peaks 1–4, 8–13, 15–18, 21, 23, 24, and 28), 8 phenolic acids and related compounds (peaks 5–7, 14, 19, 20, 22, and 29) and 7 flavonoids (peaks 25–27 and 30–33) in extracts of *L. pubiflora* by UHPLC-PDA-MS which are responsible for the biological activity. This study assessed for the first time the sedative-anxiolytic effects of *L. pubiflora* in rats besides the high resolution metabolomics analysis including the finding of pharmacologically important tropane alkaloids and glycosylated flavonoids.

## Introduction

**Figure 1:**
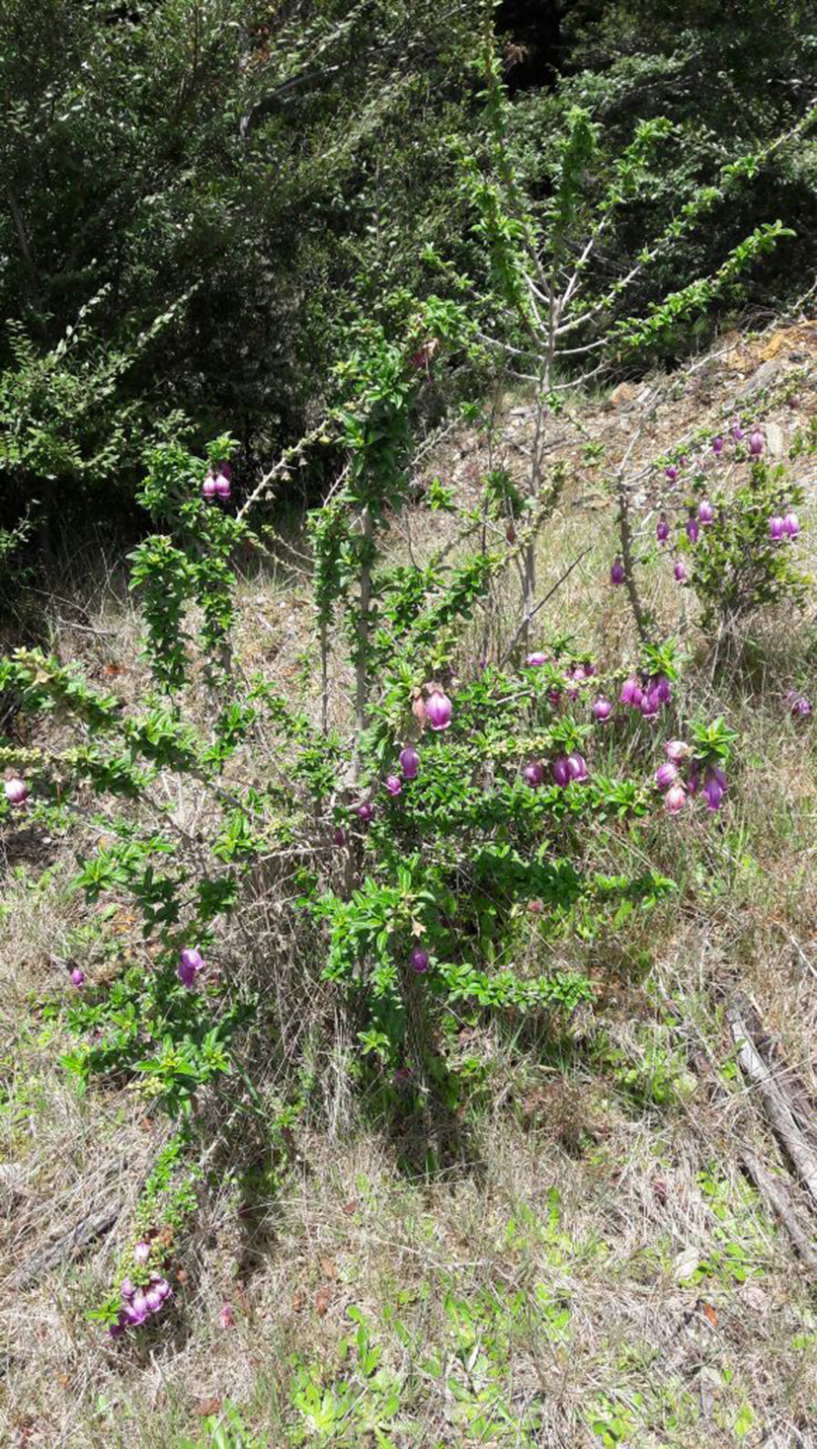
Picture of *L. pubiflora* collected on November 2011, La union, Valdivia.

Natural product's research is very important since they represent a rich source of bioactive compounds as new molecules for drug discovery and development (Atanasov et al., 2015; Waltenberger et al., 2016). However, the knowledge about locally used indigenous traditional medicines is scarce around the world, especially in Europe and America (Vogl et al., 2013; Caceres Guido et al., 2015). Some native plants in particular those belonging to the *Solanaceae* family (Ramoutsaki et al., 2002), can have strong psychoactive activities which lead to altered states of consciousness (Laderman, 1988; Bourguignon, 1989; Metzner, 1998). Their effects can be classified into hallucinogenic, stimulating, or depressing properties depending on the plant used and the secondary metabolites present in the plant. The native species *Latua pubiflora* (Griseb) Phill. (Solanaceae family, Figure 1), grows freely in Southern Chile along Cordillera de la Costa from Valdivia to Llanquihue regions (from 38°S to 43°S at 500–900 m height above sea-level). This unique species is the only member of the genus and has an important mystic role in the Mapuche indigenous culture for its putative spiritual, sedative and medicinal benefits (Olivares, 1995). In fact, the popular name of this species is “Latue” (Mapuche language) or “Palo brujo” (Spanish name), which literally means “magical stick.” This species belonging to the *Solanaceae* family is related chemo-taxonomically to the European poisonous plants *Atropa belladonna, Datura stramonium, Hyosciamus niger*, and *Mandragora* spp. used in European and Asian countries since ancient times (Hanuš et al., 2005; Beyer et al., 2009). All mentioned species contain scopolamine and atropine as main alkaloids responsible for their pharmacological properties, including sedation, pain relief and psychoactive effects (Ramoutsaki et al., 2002; Beyer et al., 2009; Soni et al., 2012). Anthropological research in Chile has shown that *Solanaceae* species were smoked in ancient rituals (Echeverria and Niemeyer, 2013; Echeverria et al., 2014; Carrasco et al., 2015) and within them *L. pubiflora* (Planella et al., 2016). This species has been consumed in different preparations (e.g., infusions, cigarettes) which can cause sedation, mouth dryness, fever, pupillary dilation, delirium, and convulsions (Olivares, 1995) depending on the doses used. *L. pubiflora* is indeed used by Mapuche medicine men to induce sedation, to reach a trance state or mystical experience and also as piscicide. Previous studies have identified the tropane alkaloids atropine and scopolamine in the fruits and leaves of this shrub (Silva and Mancinelli, 1959; Brawley and Duffield, 1972) and more recently, the presence of 3-α-cinnamoiloxytropane and apoatropine has been reported but the elucidation was tentative and the structures were reported only by using gas chromatography coupled to low resolution mass spectrometry (Muñoz and Casale, 2003). Since the herbal tea of this plant contained several alkaloids and flavonoids (Echeverria and Niemeyer, 2012), in this study two different partitions were prepared to test the alkaloids and flavonoid enriched extracts in behavioral and pharmacological tests.

In the last few years, technological advances made high resolution mass spectrometry (HR-MS) the method of choice for the rapid analysis of plants and fruits. The new ultra HPLC machines with ESI or APCI interfaces hyphenated with Q-orbitrap or Q-Tof spectrometers allow the accurate identification of several metabolites such as phenolics acids and flavonoids (Borquez et al., 2016; Cornejo et al., 2016), terpenoids (Dong et al., 2016; Simirgiotis et al., 2016b), and alkaloids (Liu C. M. et al., 2016; Liu Z. H. et al., 2016). The purpose of this study was to establish the psychopharmacological effects of enriched extracts of *L. pubiflora* by the employment of two well-established behavioral paradigms in the rodent model: the elevated plus-maze (EPM) and the pentobarbital-induced sleeping test. The EPM is a behavioral task based on the natural aversion of rodents to heights and open spaces (Pellow et al., 1985; Lister, 1990; Carobrez and Bertoglio, 2005). An increment in the number of entries and/or time spent in the open arms of the maze reflects an anxiolytic effect whereas a decrease in any of these parameters is considered an anxiogenic response (Rodgers and Cole, 1995; Hogg, 1996). Similarly, the pentobarbital-induced sleeping test is a rodent model to assess potential sedative effects (Lovell, 1986a,b). Behavioral pharmacology studies in this work were followed by an assessment of *L. pubiflora* effects on [^3^H]flunitrazepam binding by using a rat cortical membrane preparation.

In addition, and following our research program on Chilean flora metabolomics (Simirgiotis et al., 2015, 2016c; Borquez et al., 2016) we have performed the full UHPLC Orbitrap HR-MS metabolome analysis of this important native Mapuche species and related the alkaloid and flavonoids profiles with the ethnopharmacological activity. Our results provide evidence for sedative and anxiolytic properties of *L. pubiflora* and its psychoactive metabolomics for the first time.

## Materials and methods

### Drugs and reagents

Reagent grade ethanol (ETOH), methanol (MeOH), chloroform, ethyl acetate (EtOAc) and HPLC grade water were obtained from Merck KGaA, (Darmstadt, Germany). Flumazenil (Lanexat®), diazepam (Valium®) clonazepam (Klonopin™) were from Hoffmann-La Roche Inc., [^3^H]-methyl-flunitrazepam 60-85Ci/mmol were purchased from ARC American Radiolabeled Chemicals Inc. Saint Louis, MO, Trizma® Base 99.9% (Tris [Hydroxymethyl] aminomethane) Sigma-Aldrich Saint Louis, MO. HPLC standards, (rutin, quercetin, isorhamnetin, scopoletin, citric acid, kaempferol-3-O-rutinose, isorhamnetin-3-O-glucoside, atropine and scopolamine all standards with purity higher than 95% by HPLC) were purchased either from Sigma Aldrich (Saint Louis, Mo, USA), ChromaDex (Santa Ana, CA, USA), or Extrasynthèse (Genay, France).

### Plant material

The plant material used in the present study was collected during two summers (December 2010 and 2011) in a location 5 km from Parque Nacional Alerces Costero (40^0^ 10′, 22.5″ S, 73^0^ 26′ 30.5″ W), Valdivia, Chile. Aerial parts and flowers were collected and identified by the botanist Carlos Lehnebach. Leaves and twigs were air dried at room temperature and crushed to powder prior to extraction procedures. A voucher specimen was deposited at the Institute of Pharmacy (voucher number lp1052011), Universidad Austral de Chile.

### Experimental animals

Rockefeller male mice, 2 months old from the Animal House at the Instituto de Immunología, Universidad Austral de Chile (UACh) were used. They were maintained on a 12:12 light-dark schedule, in a controlled temperature (21°C), were housed in groups of 8 per polycarbonate cages and received free access to food and water. Animals were transported to the laboratory from the animal house 24 h prior to the experimental session in order to their adaptation. The experiments were achieved between 12 and 17 h. Animals were sacrificed by cervical dislocation under pentobarbital anesthesia or CO_2_ gas. All behavioral procedures were approved by ethical commission of the University Austral de Chile, UACh- DID S2001-67.

### Alkaloids-enriched extract (ALK)

In order to extract all compounds present in the plant, the plant material was extracted with acidified solvent. Thus, the alkaloid-rich extract (ALK) was obtained from 500 g of *L. pubiflora* chopped leaves using EtOH/HCl, 1% v:v (3 × 1 L) for 24 h. The extract was filtered yielding after evaporation under reduced pressure a syrupy green residue, the residue was later re-suspended in water (0.5 L). Precipitated lipids and chlorophyll were filtered off. The pH of the solution was increased to pH 10-11 using NH_4_OH and then CHCl_3_ was added (4 times × 300 mL) in order to obtain free alkaloids. The chloroform extracts were combined and evaporated under vacuum and freeze dried to yield 3.0 g of dry extract (0.6% yield w/w) which was suspended in Tween- 80 −10% for further experiments.

### Non-alkaloid (NON-ALK) extract and isolation of scopoletin

Once the alkaloids were extracted, the remaining aqueous solution was neutralized to pH 7 with NaHCO_3_ and repeatedly extracted with EtOAc (4 times × 300 mL). The extracts showed no major differences on thin-layer chromatography (TLC) and were combined and evaporated under vacuum to a small volume (50 mL). The absence of alkaloids was confirmed using the Draggendorf reaction in TLC assay and HPLC study. A portion (15 mL) was dried and suspended (0.5 g) in Tween- 80 −10% for the biological assays. The remaining concentrated solution (35 mL) was precipitated, and then filtered to yield a white amorphous powder which was crystallized on MeOH in order to obtain a pure product (65 mg). Direct comparison with a standard sample and spectroscopic analysis (IR, UV, ^1^H-NMR and mass spectra, please see Supplementary Material, Table S1) allowed us to identify the isolated compound as the coumarin scopoletin (Simirgiotis et al., 2013).

### UHPLC-PDA-HR-MS

The alkaloid and non alkaloidal partition extracts from leaves of *L. pubiflora* were analyzed for organic compounds using UHPLC coupled with high resolution mass spectrometry. Approximately 5 mg of each of the dried material dissolved in 2 mL of ultrapure water, with sonication for 2 min and filtered (PTFE filter, Merck) and 10 microliters were injected in the UHPLC instrument for UHPLC-MS analysis. A Thermo Scientific Ultimate 3000 RS UHPLC system controlled by Chromeleon 7.2 Software (Thermo Fisher Scientific, Waltham, MA, Germany) hyphenated with a Thermo high resolution Q-Exactive focus mass spectrometer (Thermo, Bremen, Germany) were used for analysis. All other conditions and parameters were set as previously reported (Simirgiotis et al., 2016b). Liquid chromatography was performed using an UHPLC C18 column (Acclaim, 150 mm × 4.6 mm ID, 2.5 μm, Thermo Fisher Scientific, Bremen, Germany) operated at 25°C. The detection wavelengths were 254, 280, and 320 nm and PDA recorded from 200 to 800 nm for peak characterization. Mobile phases were 1% formic aqueous solution (A) and acetonitrile (B). The gradient program (time (min), % B) was: (0.00, 5); (5.00, 5); (10.00, 30); (15.00, 30); (20.00, 70); (25.00, 70); and 12 min for column equilibration before each injection. The flow rate was 1.00 mL min^−1^, and the injection volume was 10 μL. Standards and extracts dissolved in methanol were kept at 10°C during storage in the auto-sampler (Simirgiotis et al., 2016c). The Spectrometer and HESI II parameters were optimized as previously reported (Simirgiotis et al., 2016c). For the quantification of atropine and scopolamine all measurements were done in triplicate (*n* = 3). For the recovery experiments of these compounds, known amounts of standards (stock solutions of 0.5 mg/mL) were added to 50 g of the plant matrix and the spiked sample processed using the same methods described above, in triplicate. The amount of the tested compounds were then measured and the percent ratio between the spiked and non-spiked (set as zero level) sample was calculated. The measured amounts in relation to the theoretically present ones were expressed as percent of recovery. The obtained recovery was 115.12 ± 1.68 and 98.3 ± 0.98% (mean ± RSD) for atropine and scopolamine, respectively.

### Grouping and dosing of animals

Rockefeller mice maintained on a 12:12 light-dark schedule, in a controlled temperature (21°C). In the behavioral assessment, all testing performed at 150 mg/kg. For the pentobarbital sleeping test, mice were divided into six groups (*n* = 10), which included three test groups treated with the different extracts with its vehicle as control. All drugs were injected intraperitoneally (i.p.). Extracts were administered 30 min prior to pentobarbital exposure 40 mg/kg. The three test groups received an injection of 150 mg/kg of the ALK extract, 150 mg/kg of the NON-ALK extract and the pure isolated compound scopoletin (7.5 mg/kg). The elevated plus-maze test (EPM) was performed using a maze of two open arms with transparent floor, two black arms enclosed by walls (6 cm × 30 cm × 10 cm) opposite to each other, joined by a central black platform (8 cm × 8 cm). Three different groups of mice were considered (10 mice each group): group one treated with diazepam, group two treated with the ALK extract and group three treated with the NON-ALK extract. In all groups the anxiolytic and locomotor activities were evaluated on the maze, after receiving flumazenil at 0.3 mg/kg s.c., 10 min prior to evaluation.

### Binding assay

[^3^H]Flunitrazepam binding assays were performed with cortical membranes from female rats of approximately 2 months old (125–150 g) obtained from Analytical Biological Services, Inc. (Wilmington, DE). The reaction was initiated by the addition of tissue (20–24 μg protein) to tubes containing 2 nM [^3^H]flunitrazepam in a final volume of 400 μL of 50 mM Tris-HCl buffer, pH 7.4. Non-specific binding was determined in the presence of 10^−4^ M clonazepam (Ortiz et al., 1999). Different concentrations of extracts were added to tubes for competition studies. Saturation binding was performed using different concentrations of [^3^H]Flunitrazepam in the presence of 1.25 × 10^−5^ g/L of *L. pubiflora* alkaloid extract. All samples were incubated at 25°C for 40 min. The assay was stopped by rapid filtration of 100 μL of each sample in 24 mm in diameter Millipore AP 40 Glass Fiber prefilters (Millipore Corporation BedFord, MA) in a Millipore manifold (Millipore Corporation BedFord, MA) followed by two - 2.5 mL ice cold buffer washes. Radioactivity of the dried filters was quantified in a Beckman LS 6000 counter with 5 mL of EcoLume scintillation cocktail. Results are shown as percentage of total binding.

### Behavioral assessment

In order to determine the experimental dose of the extract, we conducted a dose-response curve in which animals received 800 mg/kg of the ALK extract, and death was induced approximate 5 min after the injections (data not shown). Convulsions were induced at 400 mg/kg i.p. (data not shown). In contrast, 200 mg/kg of the extract induced excitatory effects. Hyperactivity was evident, which caused difficulty in the handling of mice. Therefore, we determined that 150 mg/kg i.p. produced a stable response during a 1 h observation period. Therefore, all behavioral testing was done at 150 mg/kg (i.p.).

### Pentobarbital-induced sleeping test

Mice were divided into six groups (*n* = 10), which included three test groups treated with the different extracts with its vehicle as control. All drugs were injected intraperitoneally. Extracts were administered 30 min prior to pentobarbital exposure 40 mg/kg (i.p.). The onset of sleep (or latency time, the time elapsed between injection of pentobarbital and loss of the righting reflex) and sleeping time (lapse between loss and the recovery of righting reflex) was registered according to Matsumoto et al. (1997). The three test groups received an i.p. injection of 150 mg/kg of the ALK extract, 150 mg/kg of the NON-ALK extract and 7.5 mg/kg i.p. of the isolated compound scopoletin (in the same concentration found in the ALK extract). The control groups were injected with vehicle (10% Tween 80).

### Elevated plus-maze (EPM) test

The elevated plus-maze test was performed according to Maruyama et al. (1998). It consisted of two open arms with transparent floor (6 cm × 30 cm), two black arms enclosed by walls (6 cm × 30 cm × 10 cm) arranged in a way that one pair of identical arms were opposite to each other, joined by a central black platform (8 cm × 8 cm). The apparatus was raised to a height of 40 cm above the floor and located in a room with black walls and floor, illuminated by fluorescent light (40 W). The experimental session was registered by a camera linked to a monitor and a video-recorder in an adjacent room, for further analysis by testers unaware of the treatment conditions.

Three groups of 10 mice were used, a control group (NaCl 0.9%), an extract group (150 mg/Kg of extract i.p.) and a reference group (diazepam 1 mg/Kg i,p). EPM behavior was registered 30 min after dosing. At the beginning of the test, each mouse was individually placed onto the central platform facing a closed arm, and the behavior was recorded during a 5 min session. The criterion for an arm entry was defined as four paws into a given arm. In another set of experiments: diazepam, ALK and NON-ALK treated mice received flumazenil at 0.3 mg/kg s.c., 10 min prior to evaluation of anxiolytic and locomotor activity on the maze following (Kuribara et al., 1998).

### Statistical analysis

Significance was measured using one-way ANOVA. Whenever appropriate, Student's *t*-test or χ^2^ were also employed. Data are expressed as the mean ± SEM. Statistical significance was attained at *p* ≤ 0.05. Saturation and inhibition curves were analyzed using GraphPad Prism 4 software (version 4.03, GraphPad Prism, San Diego, CA).

## Results

### Identification of secondary metabolites in *L. pubiflora*

**Figure 2:**
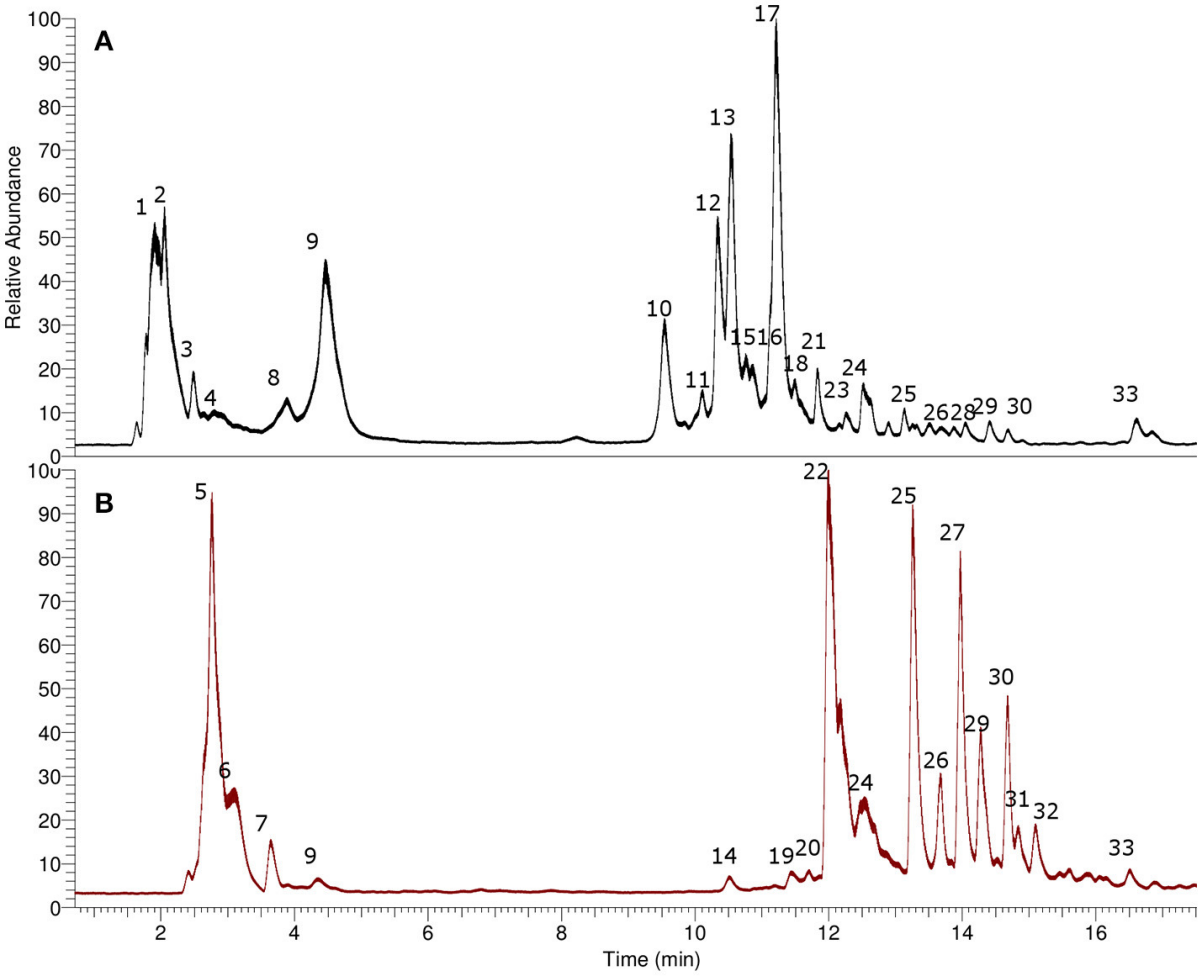
UHPLC chromatograms (TIC). **(A)** Alkaloid-rich extract, **(B)** Non alkaloid extract.

Several compounds including tropane alkaloids, phenolic acids and flavonoids were identified (Table 1) by means of high resolution mass spectrometry (UHPLC-Q-OT-MS), and the UHPLC-TIC chromatogram is depicted in Figure 2. For the tropane alkaloids the retention times of the major components were 11.21 min. for atropine, and 10.34 min. for scopolamine. The contents per g dry weight of the plant *L. pubiflora* were determined by HPLC to be 344 ± 6.32 μg for scopolamine, 1260 ± 15.24 μg for atropine and 646.8 ± 18.85 μg of scopoletin. The major tropane alkaloids -scopolamine and atropine- were present in 5.7 and 21%, respectively, in the ALK extract used for pharmacological experiments (see below). The coumarin scopoletin was isolated (see Experimental) and its structure obtained by spectroscopic methods (see Supplementary Material) and resulted the major component of the NON-ALK extract (3.90 g). Below is the detailed explanation of the accurate mass identification (Table 1). Several examples of structures and full MS spectra are shown in Supplementary Material (Figures S2).

**Table 1:** UV maxima and high resolution Q-Orbitrap MS-MS data and formulas for the metabolites identified in *L. pubiflora* extracts.

**Peak #**	**Retention time**	**Uv max**	**Tentative identification**	**Molecular formula [M-H]^−^or [M+H]^+^**	**Theoretical mass**	**Measured mass**	**Accuracy (δppm)**	**Other ions (MS-MS)**
1	1.89	–	Tropine	C_8_H_16_NO	142.12319	142.12312	−0.49	124.11265 C_8_H_14_N (deshidrotropine)
2	2.05	–	6-hydroxy-3-O-acetyl tropine	C_10_H_18_NO_3_	200.12867	200.12935	3.39	140.10771 C_8_H_14_NO (hydroxydeshydroytropine)
3	2.51	–	3-Acetamidopentoate	C_7_H_12_NO_3_	158.08227	158.08160	−4.23	
4	2.53	220-285-330	Hygrine	C_8_H_16_NO	142.12319	142.12309	−0.70	
5	2.76	235-287-345	Scopoletin^*^	C_10_H_7_O_4_	191.03498	191.03465	−1.72	
6	2.94	–	Quinic acid	C_7_H_11_O_6_	191.05611	191.05559	−2.72	
7	3.72	230	Citric acid^*^	C_6_H_7_O_7_	191.01936	191.01863	3.84	
8	3.88	–	3-O-Acetyl-tropine	C_10_H_18_NO_2_	184.13375	184.13419	2.38	124.11275, C_8_H_14_N (deshidrotropine)
9	4.46	–	7-hydroxy-3-O-acetyl tropine	C_10_H_18_NO_3_	200.12867	200.12924	2.84	140.10771 (hydroxydeshydroytropine)
10	9.54	–	Scopine	C_8_H_14_NO_2_	156.10245	156.10236	−0.57	138.09196, C_8_H_14_NO(deshydroscopine)
11	10.30	–	3α-Apotropoyloxytropane	C_17_H_22_NO_2_	272.16505	272.16617	4.11	124.11262 (C_8_H_14_N, dehydrotropane)
12	10.51	–	7-Hydroxyhyoscyamine	C_17_H_24_NO_4_	306.16998	306.17209	7.56	140.10768 (C_8_H_14_NO, dehydrated tropane)
13	10.34	–	Scopolamine^*^	C_17_H_22_NO_4_	304.15640	304.15488		156.10229 (scopine, C_8_H_14_NO_2_), 138.09167 (dehydrated scopine, C_8_H_12_NO)
14	10.55	220-285-330	Caffeoyl-glucoside^*^	C_15_H_15_O_9_	341.08781	341.08795	0.41	161.0368, 133.02886
15	10.81	–	Norhyoscyamine	C_16_H_22_NO_3_	276.15997	276.16180	6.62	
16	10.93	–	Littorine	C_17_H_22_NO_3_	276.15942	276.16092	5.42	
17	11.21	–	Atropine^*^	C_17_H_24_N O_3_	290.17758	290.17507	8.65	124.11280 (deshidrotropine, C_8_H_14_N)
18	11.24	–	6-hydroxyhyoscyamine	C_17_H_24_NO_4_	306.17209	306.16998	6.87	140.10736 (C_8_H_14_NO, dehydrated 6-hydroxy-tropane)
19	11.71	205-300-324	Feruloylquinic acid^*^	C_17_H_19_O_9_	367.10236	367.10342	2.90	191.05548, 173.08142
20	11.92	197-290-322	Feruloyl-caffeoylquinic acid	C_26_H_25_O_12_	529.13405	529.13452	0.88	173.08174
21	11.83	–	6,7-dihydroxy-3-tigloyloxytropane	C_13_H_22_NO_4_	256.15488	256.15576	3.43	
22	12.00	220-301-332	Chlorogenic acid^*^	C_16_H_17_O_9_	353.08746	353.08777	0.31	707.18060, 177.01869
23	12.45	280	6,7-dihydroxyhyoscyamine	C_17_H_24_NO_5_	322.16739	322.16490	7.72	156.10223 (C_8_H_14_NO_2_, dehydrated 6,7-hydroxy-tropane)
24	12.86	–	4,6-dihydroxyhyoscyamine	C_17_H_24_NO_5_	322.16730	322.16490	7.44	643.3393 ([2M+H]^+^ 156.10225 (C_8_H_14_NO_2_, 4,6-dihydroxy tropane)
25	13.28	256-353	Rutin^*^	C_27_H_29_O_16_	609.14611	609.14537		
26	13.54	254-365	Kaempferol-3-O-rutinose^*^	C_27_H_29_O_15_	593.15070	593.15010	1.01	285.04013 (kaempferol)
27	13.97	256-353	Isorhamnetin-3-O-glucoside^*^	C_22_H_21_O_12_	477.10385	477.10273	−2.17	315.05093 (isorhamnetin) 300.02771
28	13.14	–	6,7-dihydroxy-3-hydroxytigloyloxytropane	C_13_H_24_NO_5_	274.16545	274.16376	−6.16	
29		220-282	Aposcopolamine	C_17_H_2*o*_NO_3_	286.14432	286.14569	4.7	
30	14.68	256-353	Isorhamnetin-3-O-galactoside	C_22_H_21_O_12_	477.10385	477.10382	0.06	300.02771
31	14.72	254-354	Isorhamnetin-3-O-rhamnoside	C_22_H_21_O_11_	461.10894	461.10892	−0.04	177.01855
32	15.06	256-353	Rhamnetin-3-O-glucoside	C_22_H_21_O_12_	477.10385	477.10367	−0.37	315.05093 (rhamnetin) 300.02798, 271.02490
33	16.51	255-355	Isorhamnetin^*^	C_16_H_11_O_7_	315.05106	315.04993	3.57	

* *Compounds identified by co-elution with authentic standards*.

#### Tropane alkaloids

**Figure 3:**
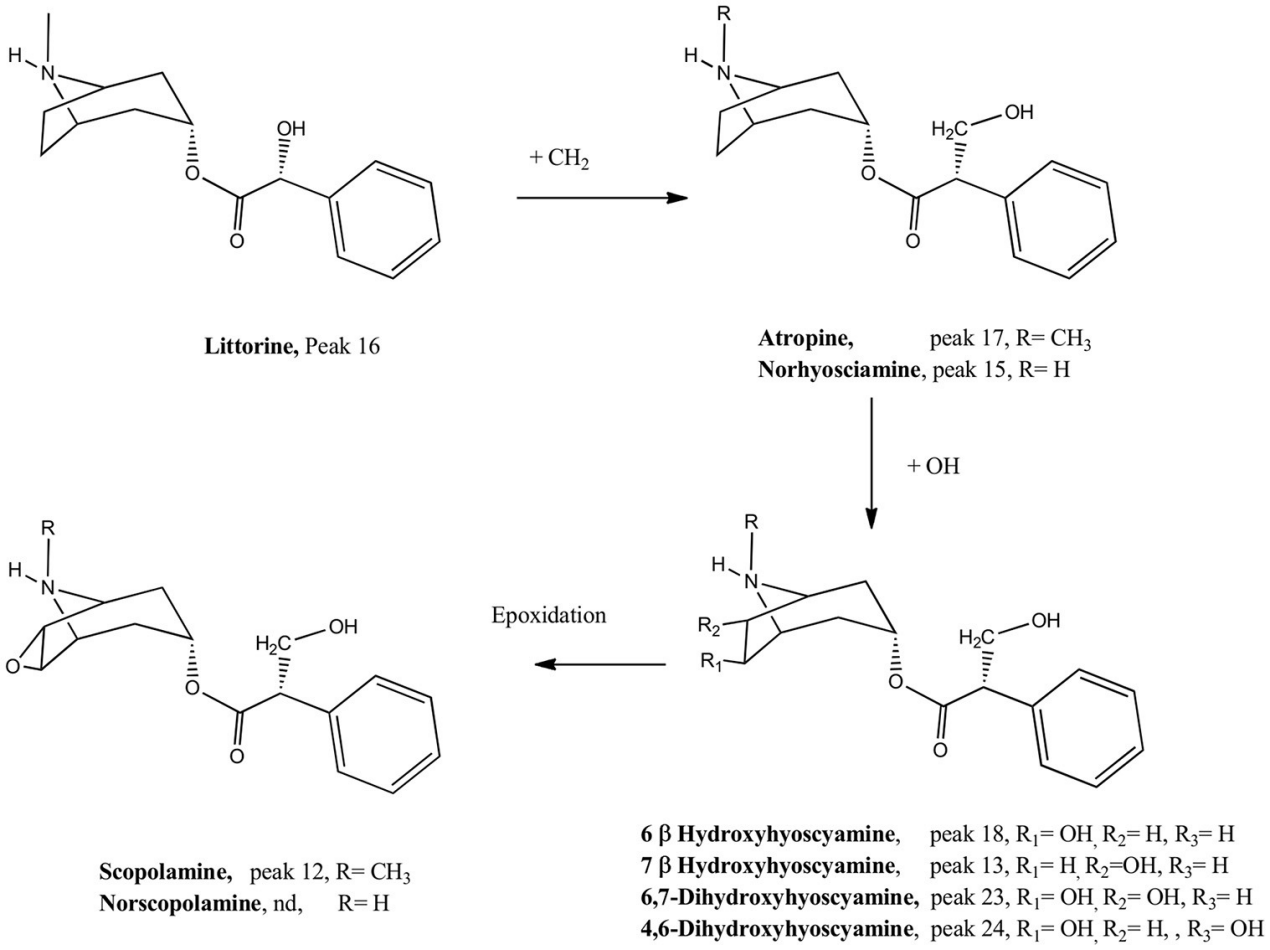
Biosynthetic relationship between the compounds detected. Nd. compound not detected.

Peak 1 with a pseudomolecular [M+H]^+^ ion at m/z: 142.12319 and showing a MS^2^ ion at m/z: 124.11265 was identified as tropine (C_8_H_16_NO) (El Bazaoui et al., 2011), while peak 2 showing an ion at m/z: 200.12867 was identified as its derivative 6-hydroxy-3-O-acetyl tropine. This identification was supported by the loss of an acetyl moiety from the parent compound at m/z: 140.10771 (C_8_H_14_NO). Peak 9 with a similar ion (200.12867) was identified as its isomer 7-hydroxy-3-O-acetyl tropine, while peak 8 was identified as 3-O-acetyl-tropine (C_10_H_18_NO_2_), this peak showing also a loss of an acetyl group at m/z: 124.11275, C_8_H_14_N (deshidrotropine). Peak 3 was identified as 3-acetamidopentoate (C_7_H_12_NO_3_) and peak 4 as the coca leaves marker hygrine (C_8_H_16_NO) (Rubio et al., 2014). Peak 10 was identified as scopine (C_8_H_14_NO_2_). This compound produced a diagnostic MS^2^ ion at 138.09196 (deshydroscopine) (El Bazaoui et al., 2011). Peak 11 was identified as 3α-apotropoyloxytropane (272.16617) (El Bazaoui et al., 2011) with MS^2^ fragment at m/z: 124.11262 (C_8_H_14_N, dehydrotropane).This compound was previously reported based on GC-MS (Muñoz and Casale, 2003) of an alkaloid extract of *L. pubiflora*. Peak 13 with a [M+H]^+^ ion at m/z: 304.15488 was identified as scopolamine (C_17_H_22_NO_4_) previously reported in this plant (Silva and Mancinelli, 1959; Brawley and Duffield, 1972) and producing a diagnostic tropane fragment at m/z: 156.10229 (scopine, C_8_H_14_NO_2_). Peaks 12 and 18 were identified as the derivatives 7-hydroxyhyoscyamine and 6-hydroxyhyoscyamine (C_17_H_24_NO_4_) yielding both a dehydrated tropane fragment at m/z: 140.10768 (C_8_H_14_NO). Peak 15 with a [M+H]^+^ ion at m/z: 276.16180 was identified as Norhyoscyamine (C_16_H_22_NO_3_) (Al Balkhi et al., 2012). Peak 16 was identified as the hyoscyamine precursor littorine (C_17_H_22_NO_3_) (Al Balkhi et al., 2012) and peak 17 as atropine (C_17_H_24_N O_3_) also previously reported (Brawley and Duffield, 1972) and producing a deshidrotropane unit at m/z: 124.11280. Figure 3 shows a biosynthetic relationship among the compounds mentioned and full MS spectra and structures is depicted in the Supplementary Material. Peak 21 was identified as 6,7-dihydroxy-3-tigloyloxytropane (C_13_H_22_NO_4_) (El Bazaoui et al., 2011). Peaks 23 and 24 were tentatively identified as the dihydroxylated derivatives of hyosciamine: 6,7-dihydroxyhyoscyamine and 4,6-dihydroxyhyoscyamine, respectively, producing both a dehydrated 6,7-hydroxy-tropane fragment at m/z: 156.10225 (C_8_H_14_NO_2_). Finally, Peaks 28 and 29 with a [M+H]^+^ ions at 274.16376 and 286.14569 were identified as 6,7-dihydroxy-3-hydroxytigloyloxytropane (C_13_H_24_NO_5_) and aposcopolamine (C_17_H_2*o*_NO_3_) respectively (El Bazaoui et al., 2011). Figure 3 shows biosynthetic relationships between the main compounds detected.

#### Phenolic acids and coumarins

Peak 5 with a [M-H]^−^ ion at m/z: 191.03465 was identified as scopoletin (C_10_H_7_O_4_) as reported (Simirgiotis et al., 2013). Peak 6 was identified as quinic acid (C_7_H_11_O_6_) and peak 7 as citric acid (C_6_H_7_O_7_). Peak 14 with a pseudomolecular anion at m/z: 341.08795 was identified as caffeoyl-glucoside (C_15_H_15_O_9_) (Simirgiotis et al., 2016b) based on UV maxima and MS fragments. Peaks 19, 20, and 22 with pseudomolecular anions at m/z: 367.10342, 529.13405, and 353.08777 were identified as the antioxidant phenolic acids: feruloylquinic acid, feruloyl-caffeoylquinic acid and chlorogenic acid, (Simirgiotis et al., 2016b) respectively.

#### Flavonoids

Peak 25 with a [M-H]^−^ ion at m/z: 609.14611 was assigned as rutin (C_27_H_29_O_16_) UV max 254–354 nm) (Brito et al., 2014) while peak 26 with a [M-H]^−^ ion at m/z 593.15010 producing a kaempferol aglycone at m/z: 285.04013 was identified as kaempferol-3-O-rutinose (C_27_H_29_O_15_, UV max: 254–365 nm) (Simirgiotis et al., 2016b). In the same manner, peaks 27, 30 and 32 all with full MS anions at around 477.10273 amu, and producing all MS^n^ fragments (315.05093, 300.02798, 271.02490 amu) diagnostics of quercetin methyl ester (rhamnetin or isorhamnetin) were identified as the isomers isorhamnetin-3-O-glucoside, isorhamnetin-3-O-galactoside and rhamnetin-3-O-glucoside (C_22_H_21_O_12_) (Simirgiotis et al., 2016a) respectively. Peak 31 with a [M-H]^−^ ion at m/z: 461.10892 was identified as isorhamnetin-3-O-rhamnoside (C_22_H_21_O_11_) (Brito et al., 2014) and peak 33 as the aglycone of the mentioned compounds isorhamnetin (C_16_H_11_O_7_).

### Sedative-hypnotic effects

**Figure 4:**
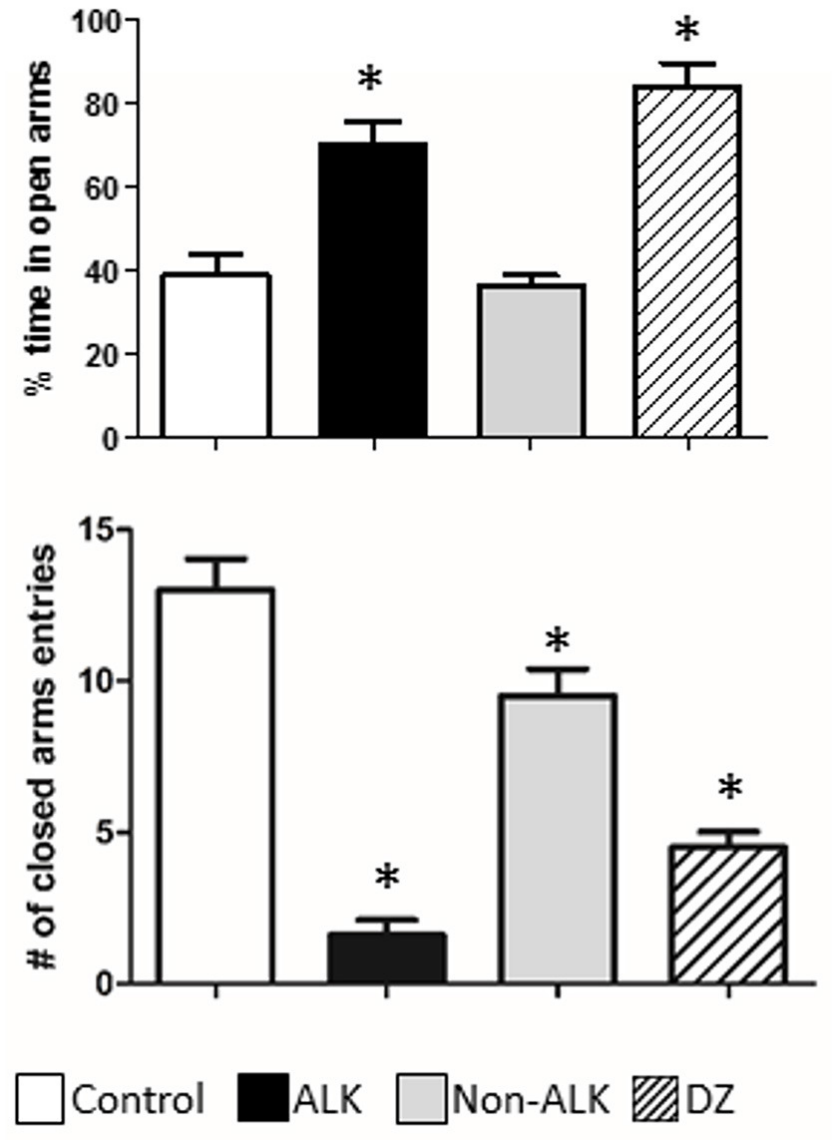
The effect of alkaloid-rich (ALK), non-alkaloid (NON-ALK) extracts, and diazepam (DZ) in the elevated plus maze. Top panel: The ALK extract and DZ 1 mg/Kg i.p. increased the percent (%) time spent in the open arms of the maze. Bottom panel: The ALK (150 mg/Kg ip), NON-ALK, and diazepam (DZ) reduced the number of closed arm entries. Data are shown as mean ± SEM, *n* = 10/group, ^*^p ≤ 0.05.

The administration of the alkaloid (ALK) extract of *L pubiflora* prior to pentobarbital injection, led to a statistically significant increase of the pentobarbital-induced sleeping time, with no modification of sleep latency (Figure 4). The non-alkaloid (NON-ALK) extract of *L pubiflora* also showed a sedative effect, increasing the sleeping time by approximately 42% compared to the control group. No changes in latency time or sleeping time were found after administration of the major coumarin isolated, scopoletin (Figure 4).

### Anxiolytic effect

**Figure 5:**
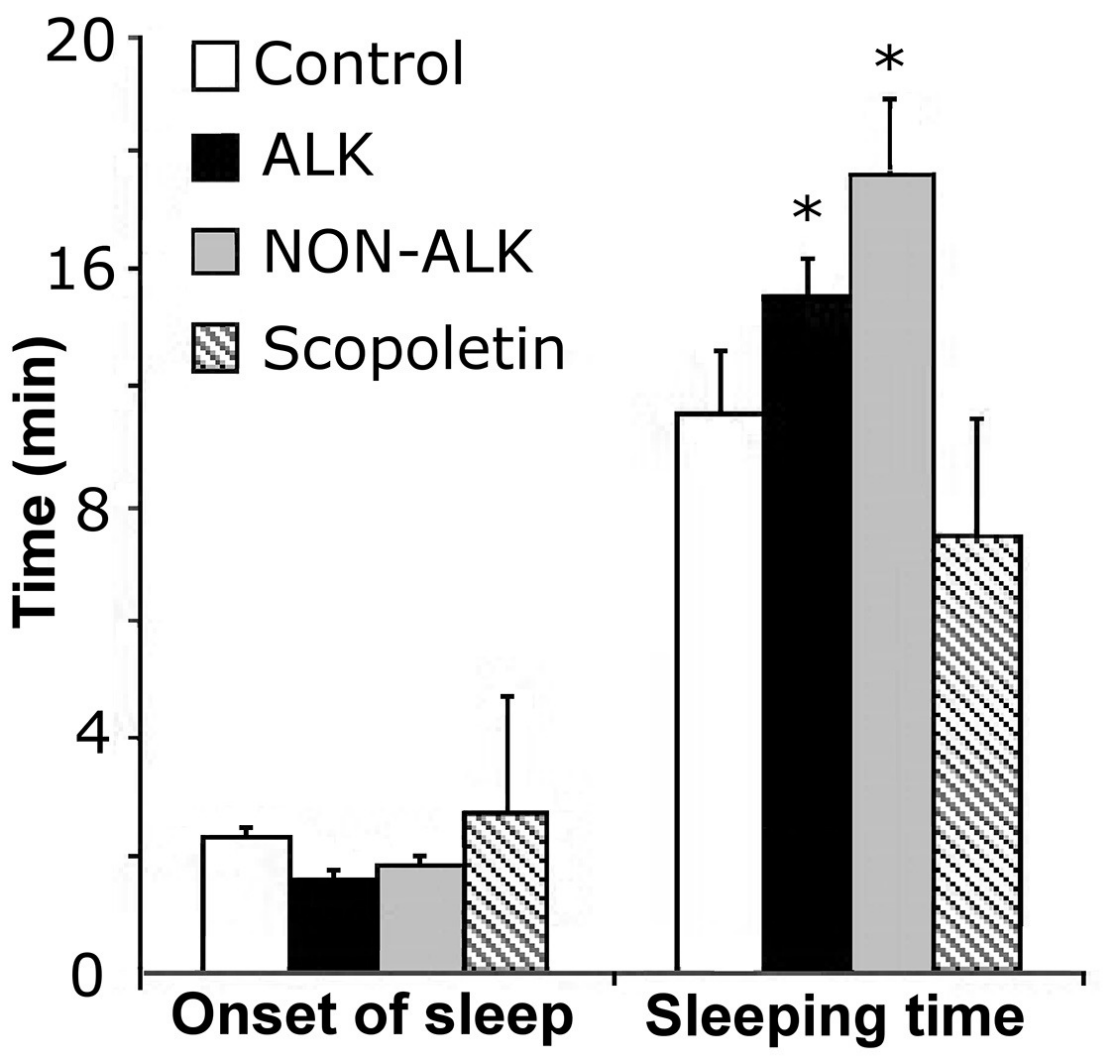
The effect of alkaloid-rich (ALK), non-alkaloid (NON-ALK) extracts, and scopoletin in the pentobarbital-induced sleeping time. The ALK, NON-ALK, and scopoletin had no significant effects in the onset of sleep. The ALK and NON-ALK extracts produced a significant increase in sleeping time when compared to control. Data are shown as mean ± SEM, *n* = 10/group, ^*^p ≤ 0.05. Onset: latency time.

**Figure 6:**
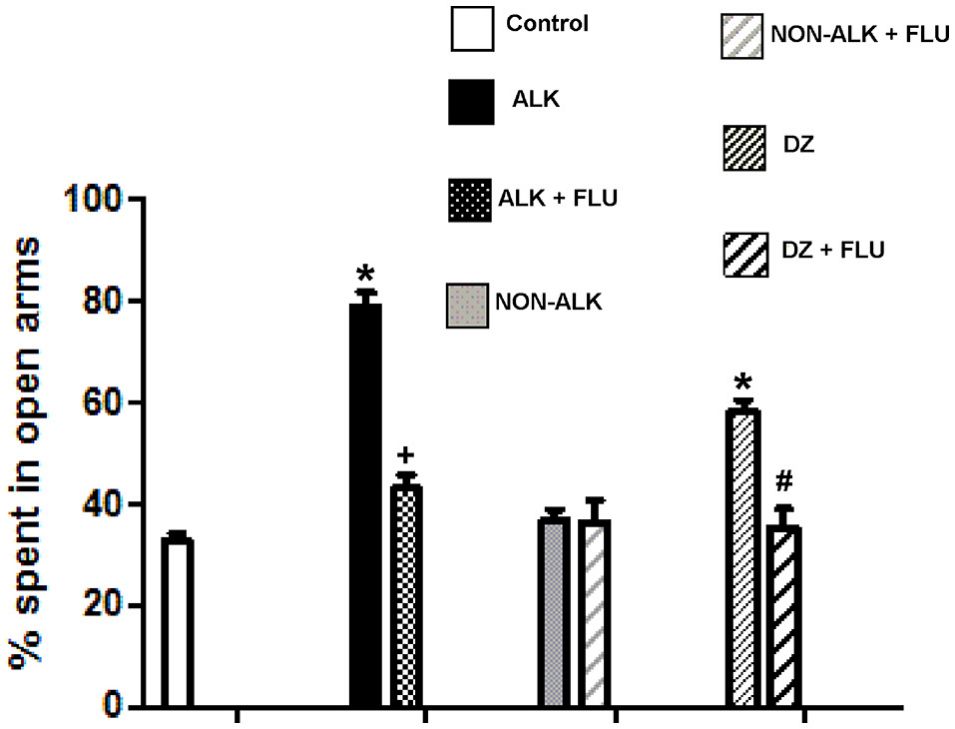
The effect of alkaloid-rich (ALK) at 150 mg/kg, non-alkaloid (NON-ALK) at 150 mg/kg extracts, and GABA_A_ receptor modulators in the elevated plus maze. The ALK (150 mg/kg i.p.) extract and diazepam 1 mg/kg i.p. (DZ) increased the % time spent in the open arms of the maze. The ALK (150 mg/kg i.p.) extract plus flumazenil 0,3 mg/Kg s.c. (ALK + FLU) reverted the increase in time spent in the open arms produced by the ALK extract. Similarly, DZ + FLU reverted the increase in time spent in the open arms produced by DZ. Data are shown as mean ± SEM, *n* = 10/group. ^*^Different from control group (*p* ≤ 0.05). ^+^ Different from ALK group (*p* ≤ 0.05). ^#^Different from DZ group (*p* ≤ 0.05). Control: Saline + Tween 20. DZ bar: Diazepam + control, DZ+FLU bar: Diazepam + flumazenil + control.

We studied the effect of the ALK and the NON-ALK extracts of *L. pubiflora* on anxiety-like behavior using the elevated plus-maze test (EPM). The animals that received the ALK extract spent significantly longer time in the open arms of the maze in comparison to that of the control group, as it was also induced by diazepam (DZ) exposure, a well-known GABA_*A*_ receptor agonist. This effect was not retained by the NON-ALK extract (Figure 5, top panel). By looking at the number of closed arm entries as an indicator of activity levels in this task, we found that the ALK, NON-ALK, and DZ injections at the selected dosages induced a decrease in this parameter (Figure 5, bottom panel). In order to confirm whether the GABA_*A*_ receptor is involved in open arm behavior, we studied the effects of flumazenil (FLU) (0.3 mg/kg s.c), a high affinity benzodiazepine receptor antagonist. Figure 6 shows that, indeed, FLU counteracted the anxiolytic effect of the alkaloid extract.

### Binding affinity to GABA_A_ receptor

**Figure 7:**
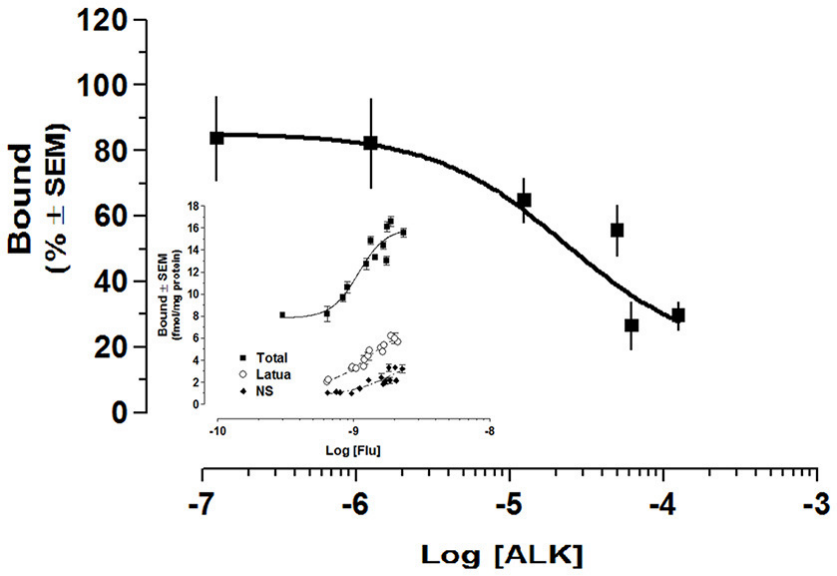
The effect of *L. pubiflora* extract on GABA_A_-receptor binding assay. Alkaloid extract of *L pubiflora* was added from 10^−7^ to 10^−4^ mg/mL in presence of 2 nM [^3^H]Flunitrazepam. The estimated IC_50_ was 2.365 × 10^−5^g/L. The saturation curve is shown in the insert, which was achieved incubating several concentrations of [^3^H] Flunitrazepam in presence of 1.25 × 10^−5^ g/L of *L. pubiflora* alkaloid extract. Each data point represents a triplicate from at least two separate experiments. ■, total binding; o, Latua (ALK); ♦, non-specific binding.

Finally, we performed receptor studies in order to determine the interactions of the alkaloid extract with GABA_A_ receptors. The alkaloid extract of *L pubiflora* was added from 10^−7^ to 10^−4^ mg/mL in presence of 2 nM [^3^H]flunitrazepam. The results of at least two independent experiments performed in triplicate showed that *L. pubiflora* ALK extract decreases flunitrazepam binding. The estimated IC_50_ was 2.365 × 10^−5^g/L (95% CI 1.6563e-005 to 3.3536e-005). It was not possible to test higher concentrations of the extract due to solubility. The saturation curve of the extract is shown in the Figure 7, which was achieved incubating several concentrations of [^3^H]Flunitrazepam in presence of 1.25 × 10^−5^ g/L of *L. pubiflora* alkaloid extract. As can be seen, the [^3^H]Flunitrazepam binding was markedly diminished in the range of concentrations that were tested. The apparent Kd could not be estimated as saturation with the extract was not observed.

## Discussion

*Latua pubiflora* has psychotropic properties and has an important mystic role in the Mapuche indigenous culture. People have suffered intoxication looking for a recreational uses of this plant (Olivares, 1995). The toxicity of *L. pubiflora* is attributed to the high content of tropane alkaloids, mainly atropine and scopolamine, by competitive inhibition of muscarinic receptors (Defrates et al., 2005; Sáiz et al., 2013). Similar effects were observed with *Datura* compounds (Steenkamp et al., 2004). In our metabolomics analysis we have identified 19 tropane alkaloids (peaks 1–4, 8–13, 15–18, 21, 23, 24, 28, and 29), 8 phenolic acids and related compounds (peaks 5–7, 14, 19, 20, 22, and 29) and 7 flavonoids (peaks 25–27 and 30–33) in extracts of *L. pubiflora* by UHPLC-PDA-MS, which are the responsible agents for the bioactivity. Both extracts, the alkaloid and non-alkaloid extracts of this plant increased the pentobarbital-induced sleeping time. However, we found that the alkaloid extract of this plant induced an anxiolytic effect in the EPM task, an effect that it could not only be explained by the presence of the tropane derivatives; since scopolamine induces an anxiogenic effect in rats (Rodgers and Cole, 1995; Smythe et al., 1996). In addition, our results have shown that both types of extracts exhibited a reduced locomotor activity in the EPM, suggesting that other mechanisms could be involved. In this regard, the inhibitory GABA_A_ receptor complex is the major target for the more prescribed anxiolytic medicines: the benzodiazepines, which also impaired locomotion by causing ataxia as an important adverse effect. Benzodiazepines act as positive allosteric modulators of GABA_A_ receptor, enhancing chloride influx leading to hyperpolarization, for binding to specific pocket on the receptor. Based on this observations, and the evidence that diverse natural products as valerenic acid (Benke et al., 2009) and honokiol (Bernaskova et al., 2015) have shown GABAergic activities, we conducted EPM in the presence of flumazenil, a benzodiazepine antagonist. The partial reversion observed in the open arm-behavior of the ALK-treated mice suggested that the GABA_A_ receptor can mediate, at least in part, these effects. These effects could be attributed to a synergic combination of some of the alkaloids detected: norhyoscyamine, 6-hydroxy-3-O-acetyl-tropine, hygrine, 3-O-acetyl-tropine, 7-hydroxy-3-O-acetyl-tropine, scopine, 3-Apotropoyloxytropane and 7-hydroxyhyoscyamine, besides scopolamine and atropine the most abundant ones. The GABA_A_ receptor complex has more than 10 modulatory binding sites generated by different assembly of its five subunits (Rudolph and Möhler, 2006). Displacement of the [^3^H] Flunitrazepam binding by the ALK-extract of *L. pubiflora* to brain membranes is suggestive of the presence of compound(s) present in this extract that compete with the benzodiazepine site at GABA_A_ receptors. we propose that such displacement could reflects subtype specificity (anxiolytic α2/3βγ2) mediated by alkaloid compounds present in this extract or at least, some of them (compounds 1–4, 8–13, 15–18, 21, 23, 24, 28, and 29) can act on the GABA_A_ receptor complex. In addition to those alkaloid compounds, we found several phenolic acids (peaks 14, 19, 20, and 22) and flavonoids (peaks 25–27, 30–32, and 33). These non-alkaloid compounds could be the responsible for the sedative but no anxiolytic activity of the NON-ALK extract because they showed no effect on the EPM. For better insight of specific GABAergic mechanism, more selective methodologies are required, such as the targeting gene encoding the different subunits, or using selective subtype GABAA receptor antagonists (Chagraoui et al., 2016). Those experiments could be interesting to carry out provided we have several isolated candidate molecules for anxiolytic actions from this plant.

In addition to those alkaloid compounds, we isolated and tested the main non-alkaloid compound isolated: scopoletin, which had been identified as an anti-inflammatory agent of *Morinda citrifolia* (Wang et al., 2002). More recently, scopoletin has been shown to suppress the release of PGE2 and pro-inflammatory cytokines (Kim et al., 2005). Furthermore, several pharmacologic activities have been reported for scopoletin, including antimicrobial, antinociceptive, and hypotensive (Farah and Samuelson, 1992; Erazo et al., 1997). Scopoletin has also been proposed to be involved in the neuropathy induced by Cassava (Ezeanyika et al., 1999). More research is needed to support our findings (isolation of all compounds and testing) but this study is a starting point on the metabolomics and bioactivity of this unique Mapuche species.

## Conclusion

The plant showed a plethora of bioactive compounds that were analyzed using high-resolution mass spectrometry, including important alkaloids and flavonoids. The extracts produced an increase of sleeping time and alteration of motor activity in mice that could be in part, attributed to the content of tropane alkaloids, largely known by their antagonist activity on muscarinic receptors. We found that *L. pubiflora* exhibits sedative and anxiolytic effects that may also be mediated through GABA_A_ receptors. These findings can explain the use of this plant in traditional medicine and religious or mystical ceremonies practiced by Mapuche people.

## Author contributions

ES and JO designed the pharmacological experiments, while MR and ES performed the pharmacological tests in rats. JP performed ^1^HNMR experiments of scopoletin and other spectroscopy experiments and wrote the table. JN and JP prepared the extracts from the plant and isolated scopoletin. JJ performed data analysis and revised data for the manuscript. JB and MS performed the LC-MS analysis. MS, AM, and ES wrote and revised the manuscript.

### Conflict of interest statement

The authors declare that the research was conducted in the absence of any commercial or financial relationships that could be construed as a potential conflict of interest.

## Abbreviations

UHPLC: Ultra-high performance liquid chromatography
PDA: Photodiode array detector
GABA: Gamma aminobutyric acid
ALK: alkaloid
HESI: heated electrospray
APCI: Atmospheric pressure chemical ionization.
