# Supplementary material for: High Resolution UHPLC-MS Metabolomics and Sedative-Anxiolytic Effects of Latua pubiflora: A Mystic Plant used by Mapuche Amerindians
Source: Front Pharmacol. 2017 Jul 26;8:494. doi: 10.3389/fphar.2017.00494 (PMC5527703; doi:10.3389/fphar.2017.00494)

**High resolution UHPLC-MS metabolomics and sedative-anxiolytic effects of *Latua pubiflora*: a mystic plant used by Mapuche amerindians**

Eliana L. Sánchez-Montoya^1^, Marco A. Reyes^1^, Joel Pardo^1^, Juana Nuñez-Alarcón^2^, José G. Ortiz^3^, Juan Carlos Jorge^4^, Jorge Bórquez^5^, Andrei Mocan^6,7*^, Mario J. Simirgiotis^1,8^*

^1^Instituto de Farmacia, Facultad de Ciencias, Universidad Austral de Chile, Valdivia, Chile.

^2^Instituto de Química, Facultad de Ciencias, Universidad Austral de Chile, Valdivia, Chile.

^3^Department of Pharmacology, Medical Sciences Campus, University of Puerto Rico, San Juan, Puerto Rico

^4^Department of Anatomy, Medical Sciences Campus, University of Puerto Rico, San Juan, Puerto Rico ^5^Laboratorio de productos Naturales, Departamento de Química, Facultad de Ciencias Básicas, Universidad de Antofagasta, Chile

^6^Department of Pharmaceutical Botany, Faculty of Pharmacy, University of Medicine and Pharmacy “Iuliu Hațieganu”, Ghe. Marinescu 23, 400337 Cluj-Napoca, Romania

^7^ICHAT and Institute for Life Sciences, University of Agricultural Sciences and Veterinary Medicine, Calea Mănăştur 3-5, 400372 Cluj-Napoca, Romania

^8^ Center for Interdisciplinary Studies on the Nervous System (CISNe), Universidad Austral de Chile, Valdivia, Chile.

*Corresponding authors:

Dr. Andrei Mocan

Department of Pharmaceutical Botany, Faculty of Pharmacy, University of Medicine and Pharmacy “Iuliu Hațieganu”, Ghe. Marinescu 23, 400337 Cluj-Napoca, Romania

Email: mocan.andrei@umfcluj.ro

Dr. Mario J. Simirgiotis

Instituto de Farmacia. Facultad de Ciencias. Center for Interdisciplinary Studies on the Nervous System (CISNe), Universidad Austral de Chile

E-mail: mario.simirgiotis@uach.cl

**Table S1.** Spectroscopic data for scopoletin obtained from NON-ALK extract

IR: (vmax cm^-1^) 3340 (phenol), 1707, (carbonyl), 3106, 3031, 1628, 924 (aromatic)

UV: (λ max: nm MeOH) 238, 243, 251sh, 2969, 343, ALCl_3_: 228, 254, 261sh, 294, 344.

ALCl_3_ + HCl: 228, 253, 297, 345. NaOAc: 233sh, 277, 295sh, 392.

NaOAc + H_3_BO_3_: 246sh, 300, 346.

1H NMR: (200 MHz, CDCl_3_: 7.6 (H-3), 6.92 (H-5), 6.85 (H-8), 6.27 (H-4), 6.18 (OH), 3.96 (OCH_3_).

HR Mass: (HESI Q-orbitrap, *m/z -*) 191.03465, 116 (M-H-H_2_O), 163 (M-H-H_2_O-CH_3_) 148 (M-H-CH_3_CO)

**Figure S2**. (a-s) Full MS spectra and structures for peaks 1, 2, 4, 8-18, 21, 24, 26, 27, 29, and 31.


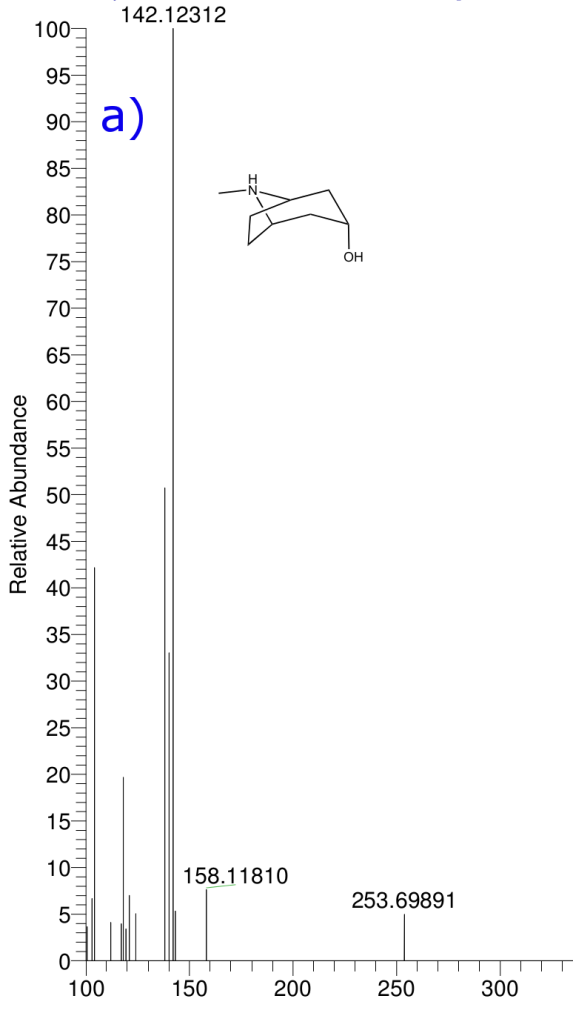

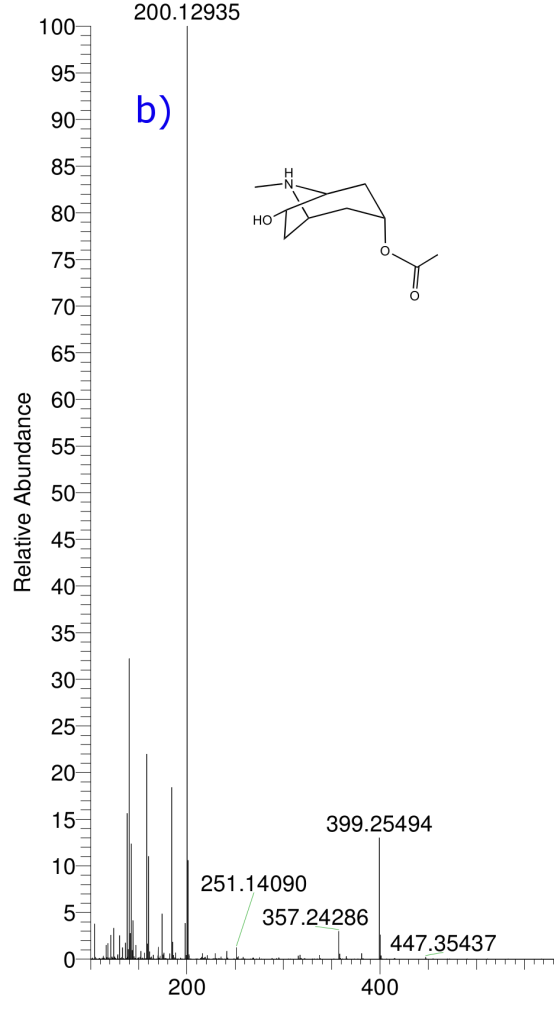


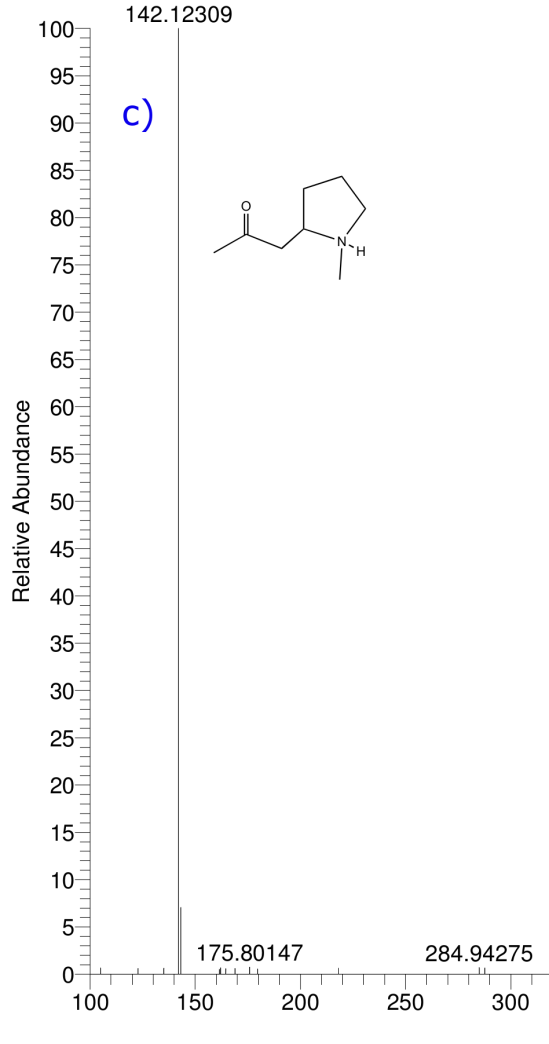

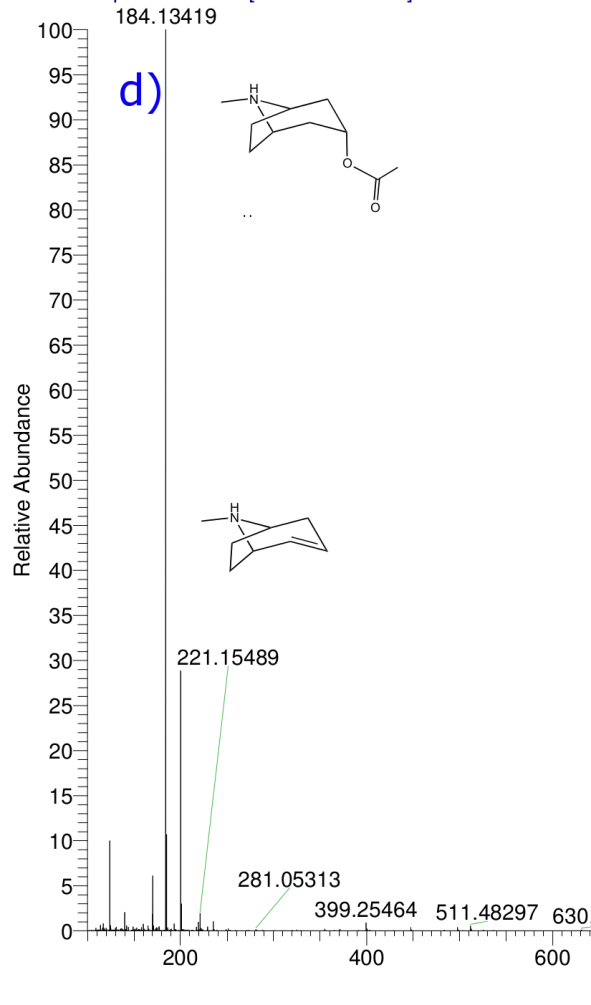

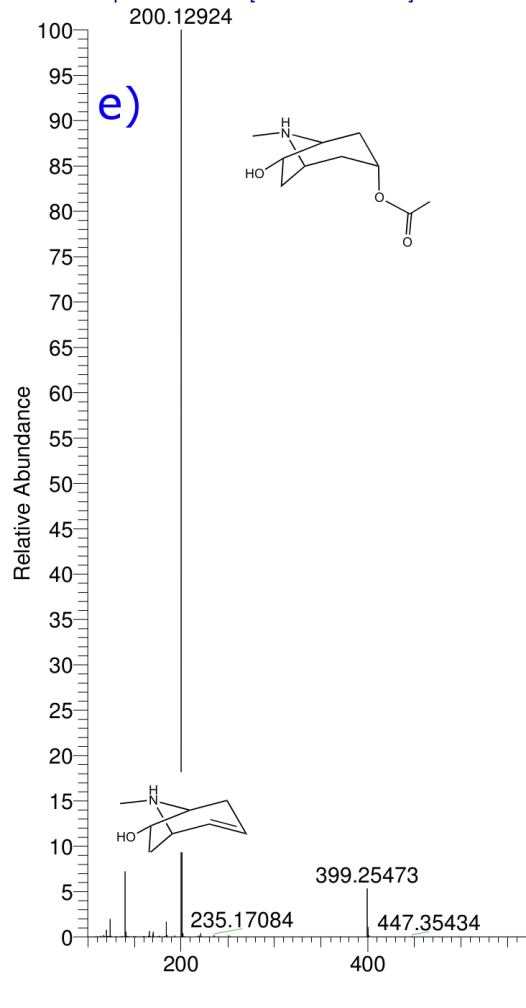

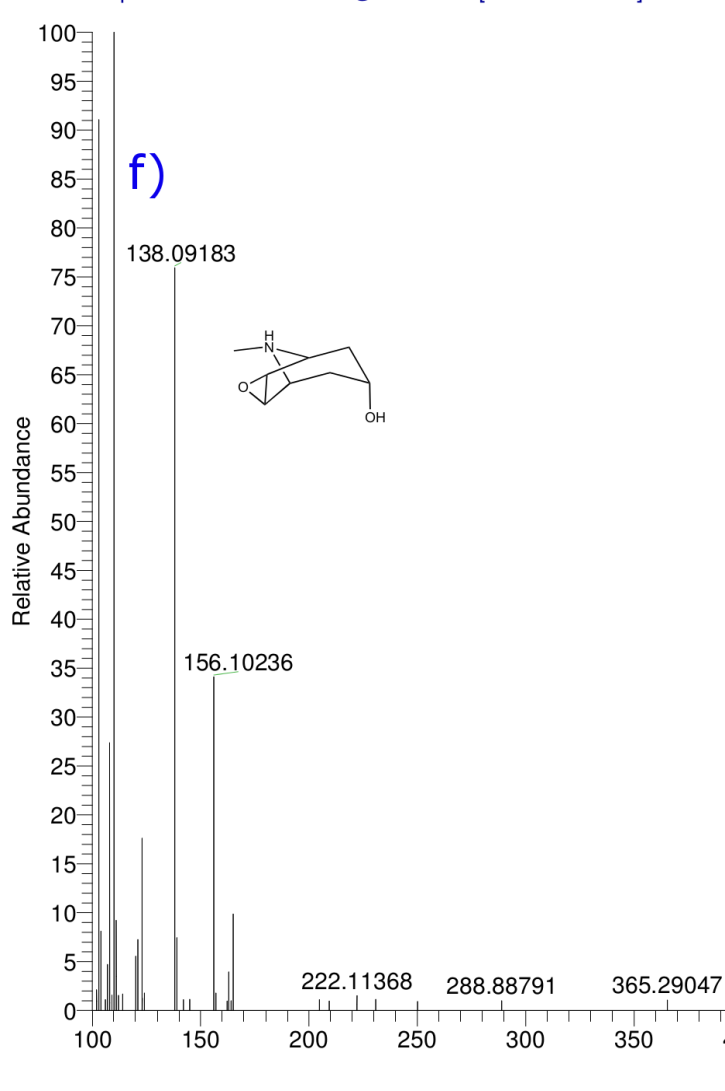

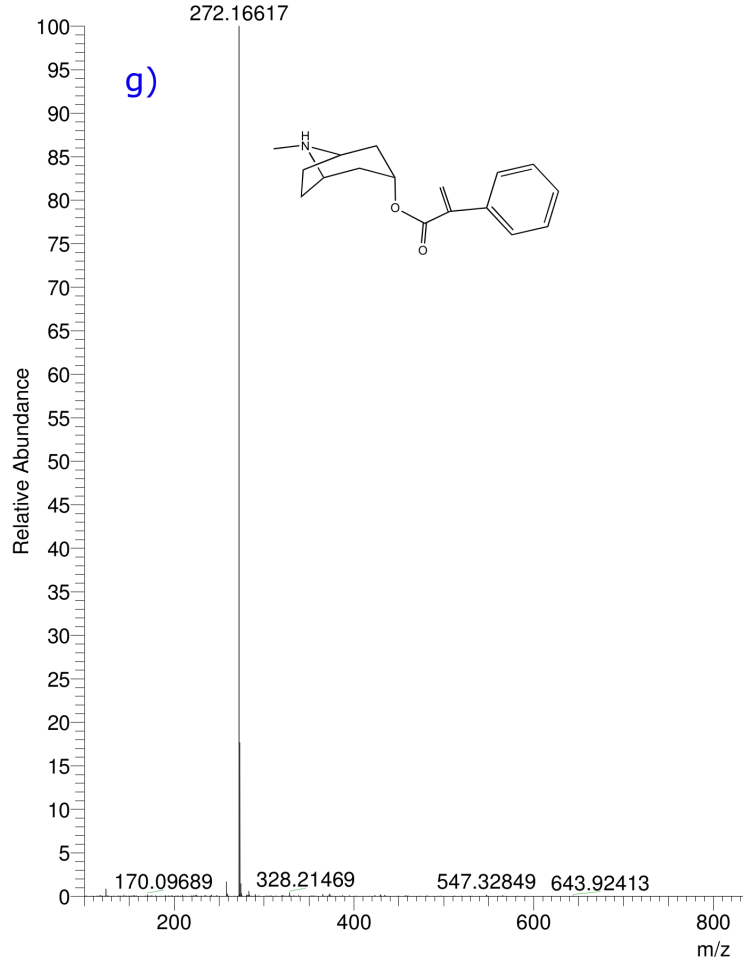


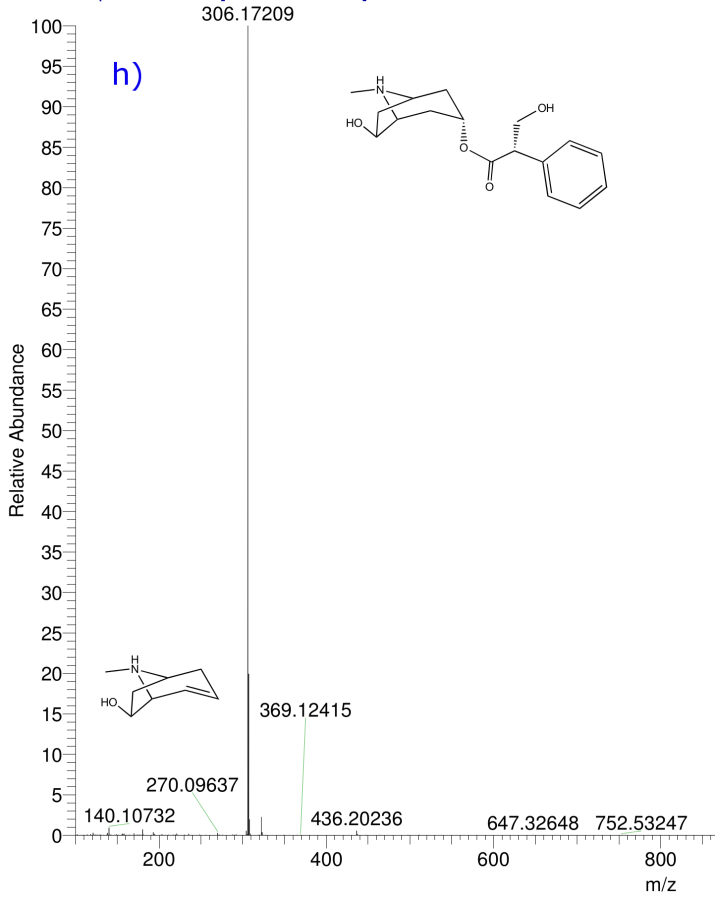


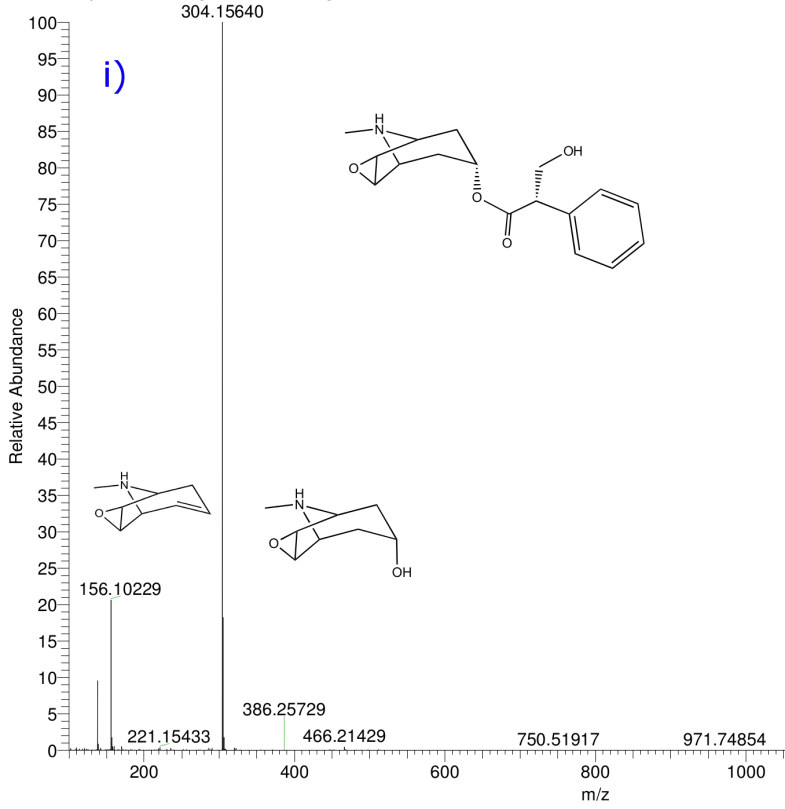


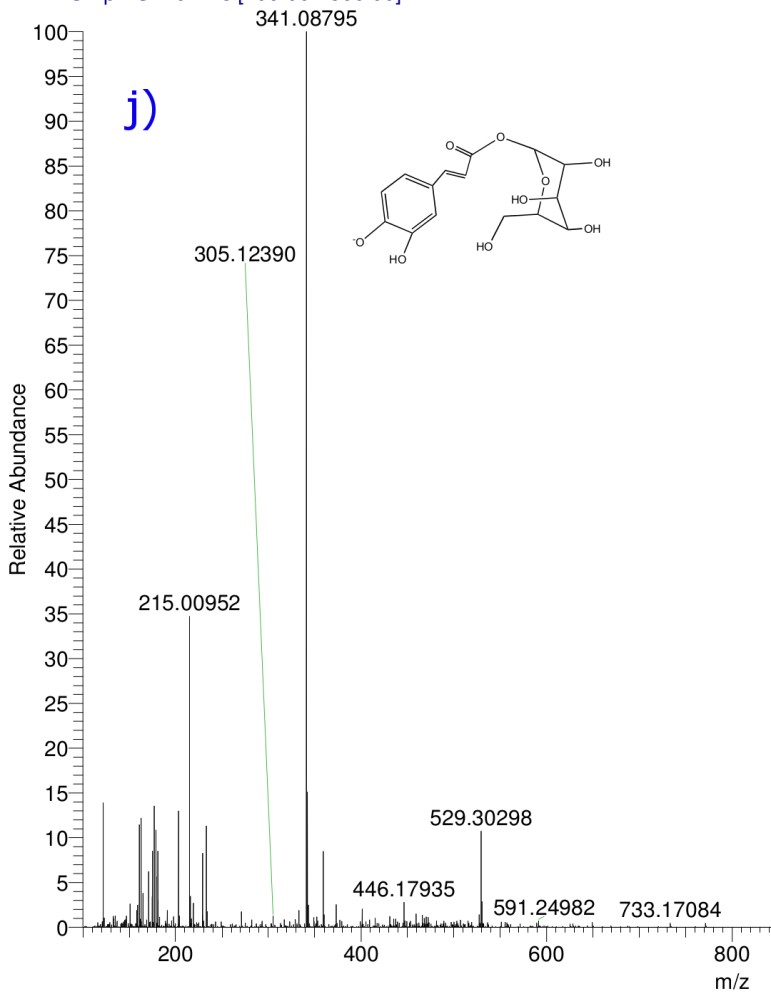

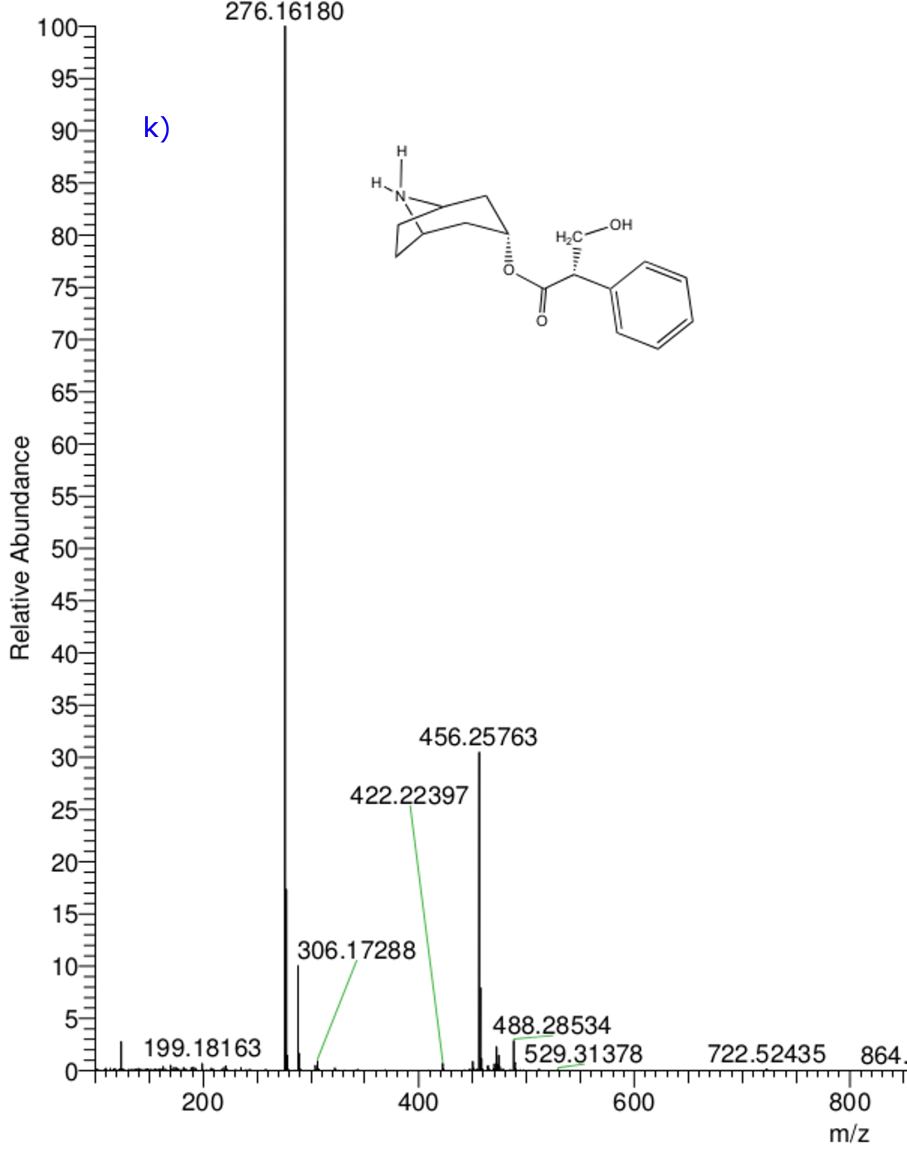

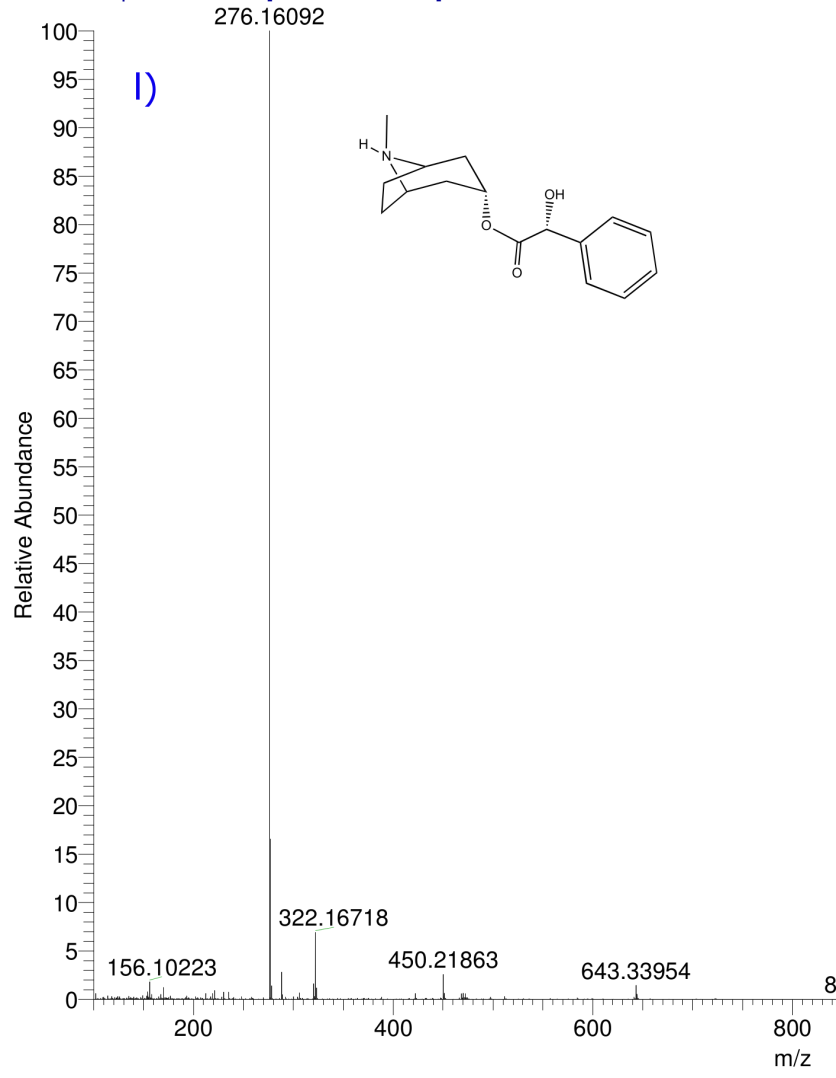


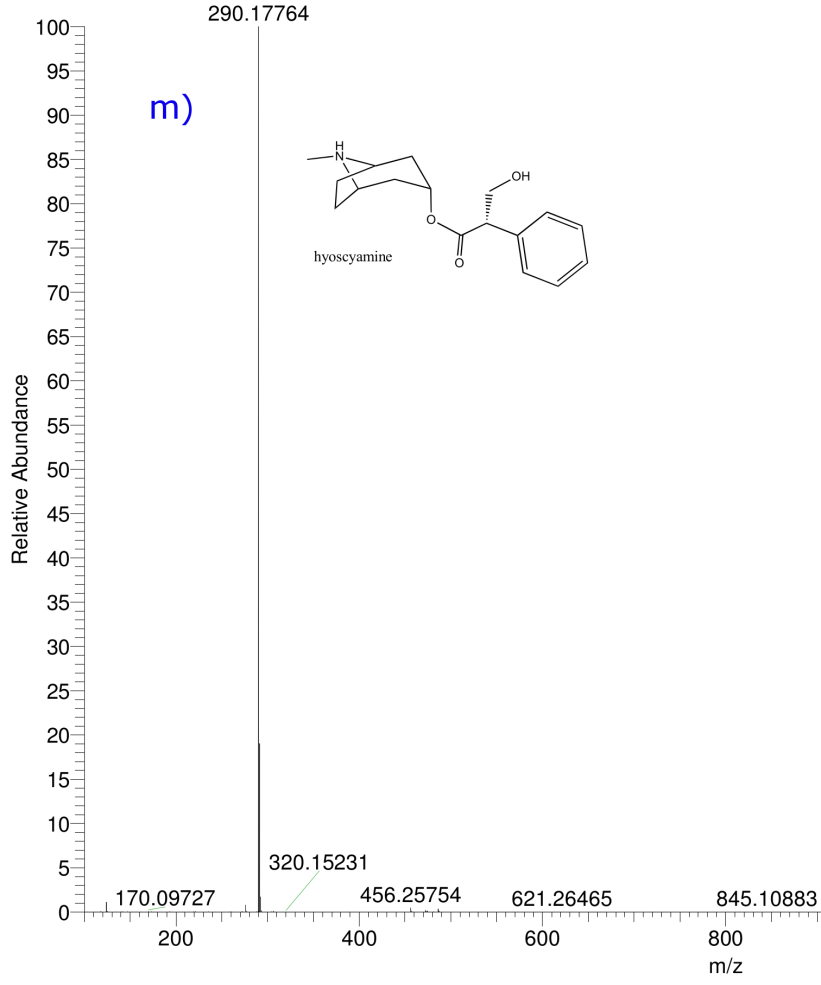

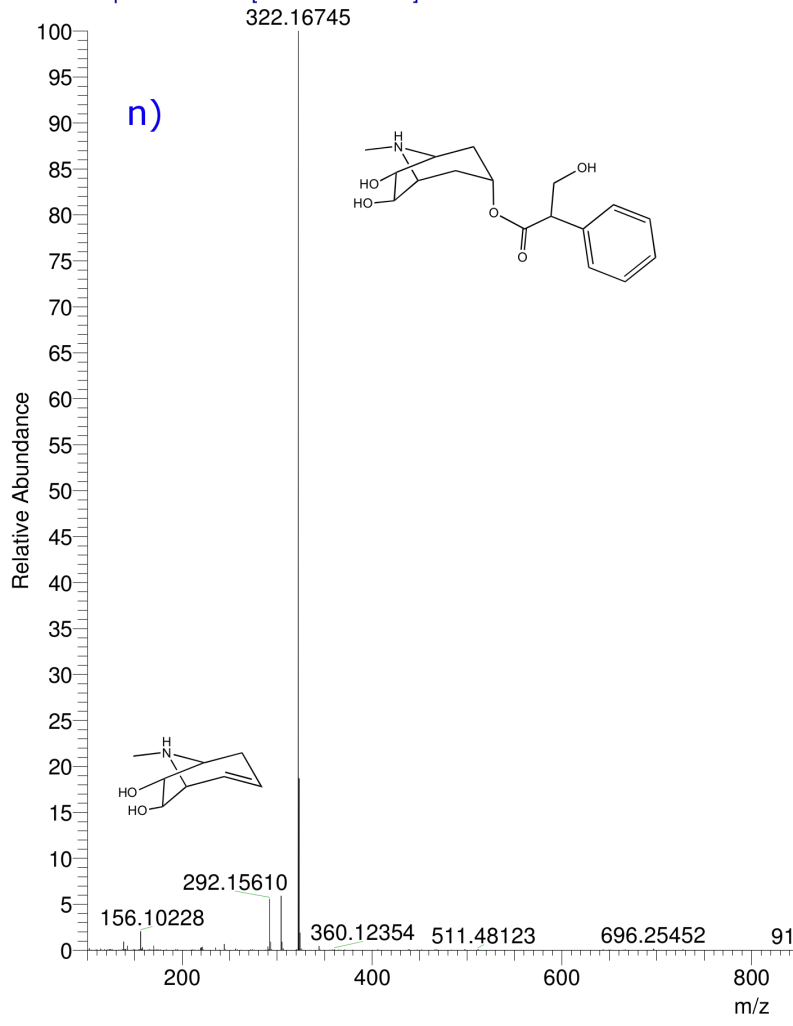

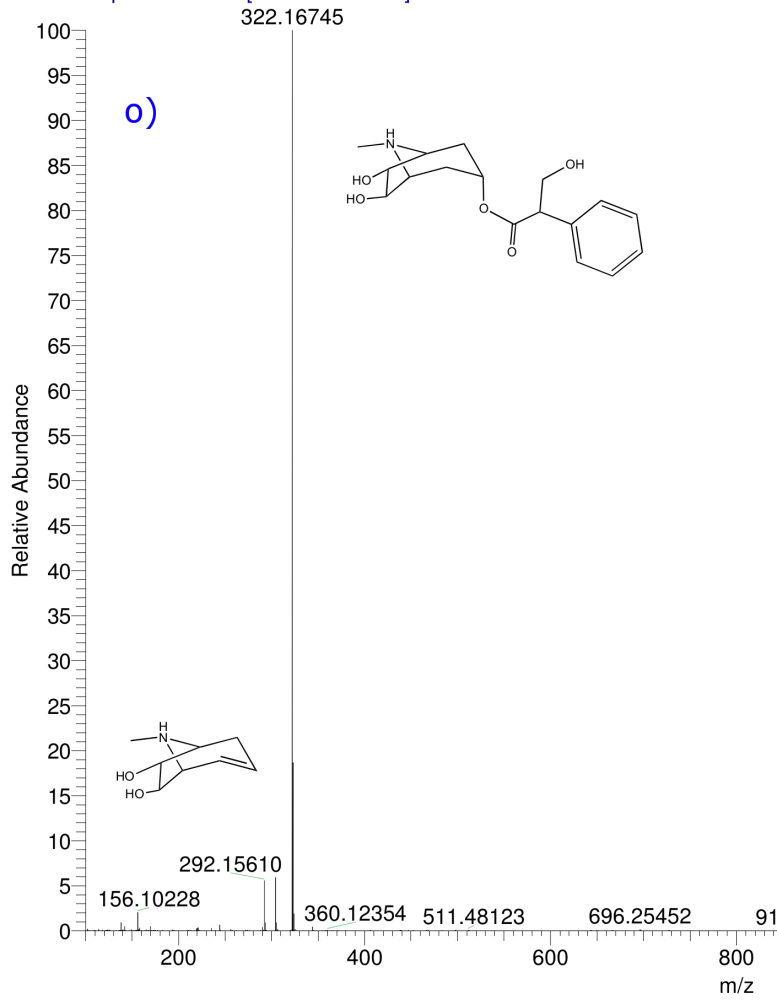

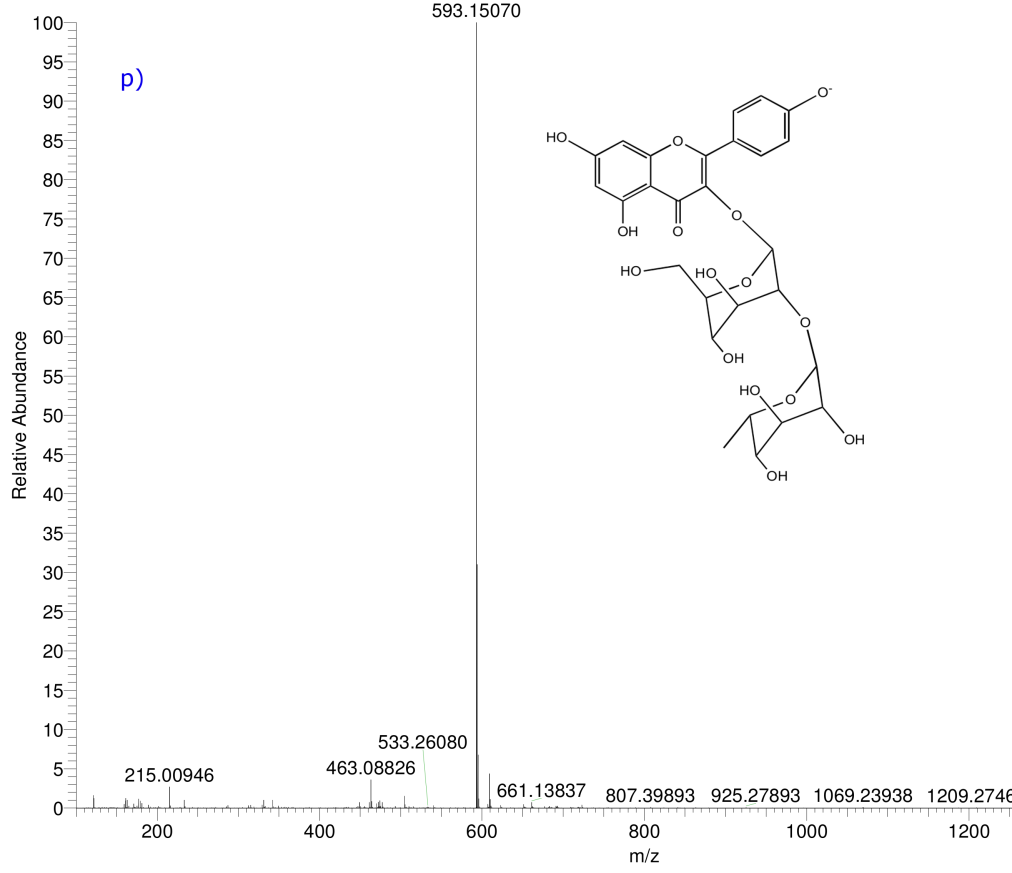

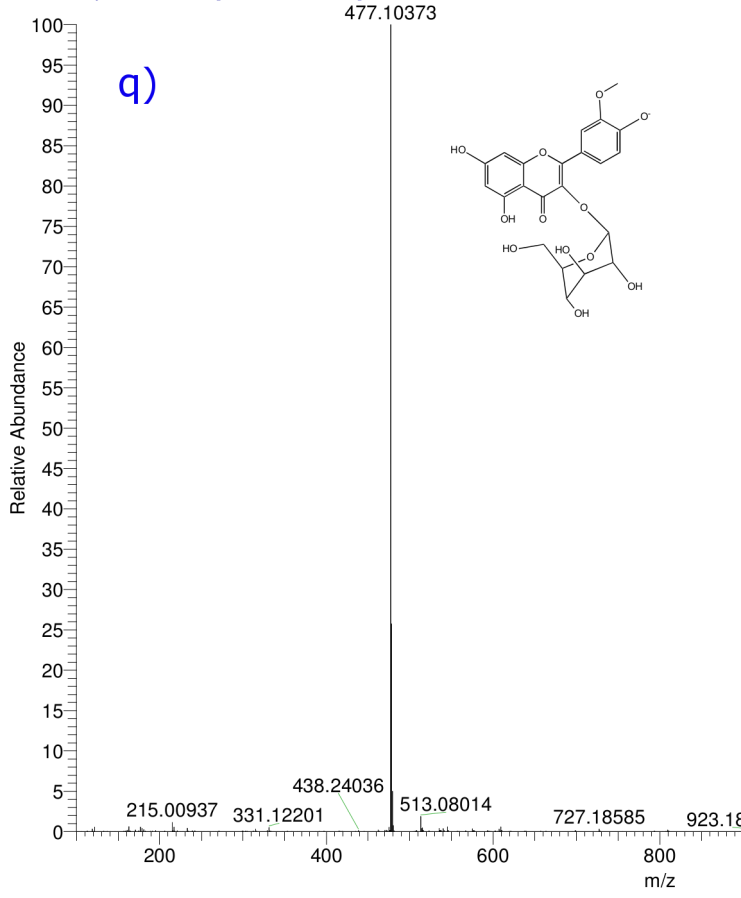

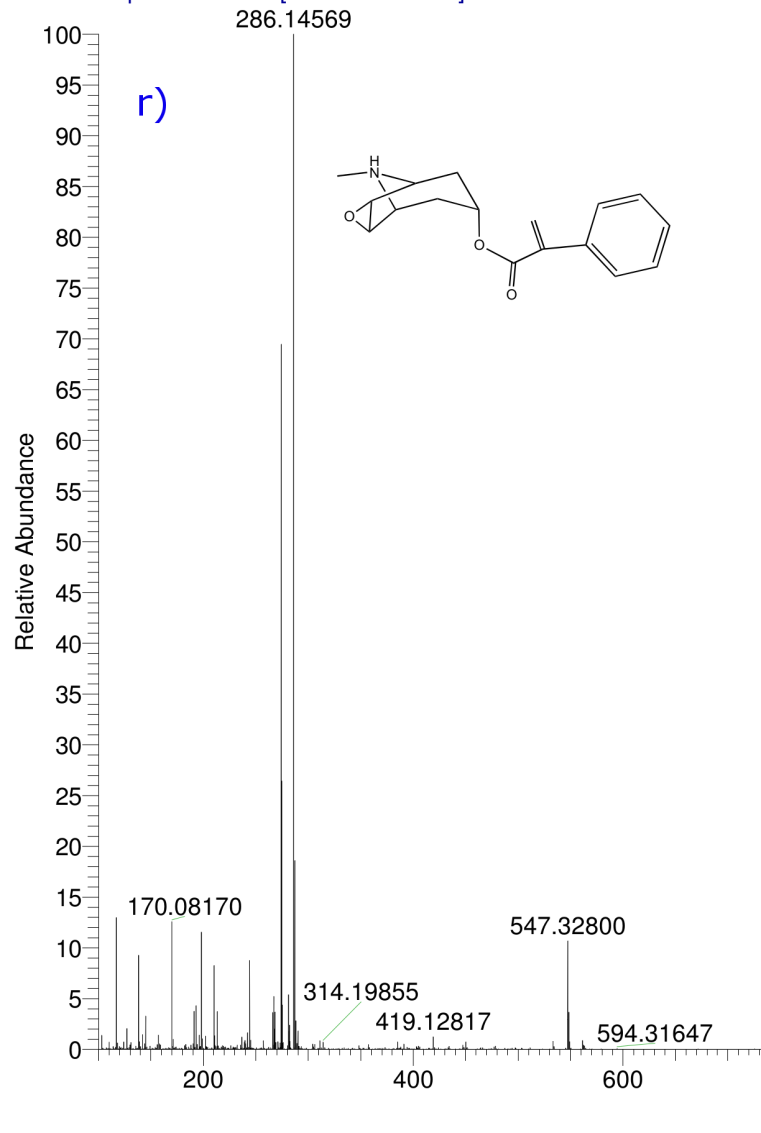

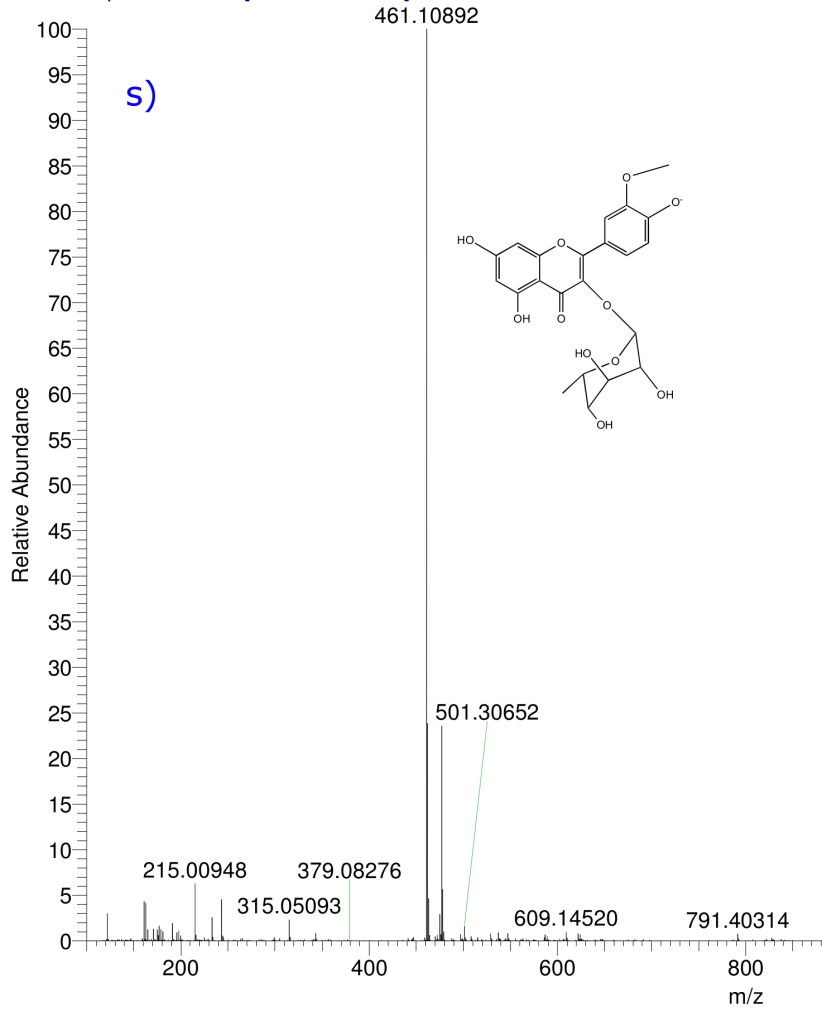

Supplement: Supplementary file 1 [file Table1.docx]
